# NR4A1 and NR4A2 orphan nuclear receptors regulate endothelial-to-hematopoietic transition in mouse hematopoietic stem cell specification

**DOI:** 10.1242/dev.201957

**Published:** 2024-11-26

**Authors:** Diana Sá da Bandeira, Chris D. Nevitt, Felipe Segato Dezem, Maycon Marção, Yutian Liu, Zakiya Kelley, Hannah DuBose, Ashley Chabot, Trent Hall, Claire Caprio, Victoria Okhomina, Guolian Kang, Jasmine Plummer, Shannon McKinney-Freeman, Wilson K. Clements, Miguel Ganuza

**Affiliations:** ^1^ Department of Hematology, St. Jude Children's Research Hospital, Memphis, TN 38105, USA; ^2^ Department of Developmental Neurobiology, St. Jude Children's Research Hospital, Memphis, TN 38105, USA; ^3^ Department of Biostatistics, St. Jude Children's Research Hospital, Memphis, TN 38105, USA; ^4^ Centre for Haemato-Oncology, Barts Cancer Institute, Queen Mary University of London, London EC1M 6BQ, UK

**Keywords:** AGM, Developmental hematopoiesis, Endothelial to hematopoietic transition, HSC specification, Hematopoietic stem cells, NR4A

## Abstract

Hematopoietic stem cells (HSCs) sustain life-long hematopoiesis and emerge during mid-gestation from hemogenic endothelial progenitors via an endothelial-to-hematopoietic transition (EHT). The full scope of molecular mechanisms governing this process remains unclear. The NR4A subfamily of orphan nuclear receptors act as tumor suppressors in myeloid leukemogenesis and have never been implicated in HSC specification. Here, we report that *Nr4a1* and *Nr4a2* expression is upregulated in hemogenic endothelium during EHT. Progressive genetic ablation of Nr4a gene dosage results in a gradual decrease in numbers of nascent c-Kit^+^ hematopoietic progenitors in developing embryos, c-Kit^+^ cell cluster size in the dorsal aorta, and a block in HSC maturation, revealed by an accumulation of pro-HSCs and pre-HSC-type I cells and decreased numbers of pre-HSC-type II cells. Consistent with these observations, cells isolated from embryonic day 11.5 *Nr4a1^−/−^*; *Nr4a2^−/−^* aorta-gonads-mesonephros are devoid of *in vivo* long-term hematopoietic repopulating potential. Molecularly, employing spatial transcriptomic analysis we determined that the genetic ablation of *Nr4a1* and *Nr4a2* prevents Notch signaling from being downregulated in intra-aortic clusters and thus for pro-HSCs to mature into HSCs. Interestingly, this defect is partially rescued by *ex vivo* culture of dissected aorta-gonads-mesonephros with SCF, IL3 and FLT3L, which may bypass Notch-dependent regulation. Overall, our data reveal a role for the NR4A family of orphan nuclear receptors in EHT.

## Teaser

**Summary:** Mouse *Nr4a1*^−*/*−^*Nr4a2*^−*/*−^ embryos lack hematopoietic stem cell activity at E11.5, implicating NR4A orphan nuclear receptors in endothelial-to-hematopoietic transition, a key step in in hematopoietic stem cell specification.

## INTRODUCTION

Hematopoietic stem cells (HSCs) are a paradigm for the immense clinical value of stem cells. HSCs have been extensively exploited to treat hematological diseases due to their capacity for life-long replenishment of the blood following transplantation (Blau and Daley, 2019). Despite extensive efforts (Lis et al., 2017; Sugimura et al., 2017), robust *in vitro* expansion of HSCs remains an unmet clinical need. Further understanding of the molecular regulation of HSC ontogeny has the potential to circumvent this stalemate, as HSCs expand significantly during embryogenesis and early neonatal life (Medvinsky et al., 2011; Mikkola and Orkin, 2006).

In mammals, hematopoiesis emerges in at least three waves during development (Medvinsky et al., 2011). The first two waves originate in the yolk sac and generate primitive erythrocytes and definitive erythroid-myeloid progenitors, which support the growing embryo and contribute very little to adult hematopoiesis (Medvinsky et al., 2011; Mikkola and Orkin, 2006). A third wave of development with an intra-embryonic origin yields multi-lineage HSCs with the ability to contribute to life-long hematopoiesis (Medvinsky et al., 2011; Mikkola and Orkin, 2006). In mice, transplantable HSCs are first detected at day 10.5 of embryonic development (E10.5) (Gekas et al., 2005; Kumaravelu et al., 2002; Medvinsky and Dzierzak, 1996; Medvinsky et al., 2011; Muller et al., 1994). HSC emergence is observed in the aortas of zebrafish as *runx1^+^* cells around 24 h post-fertilization (hpf) (Bertrand et al., 2010a; Bonkhofer et al., 2019; Gering and Patient, 2005; Tamplin et al., 2015) and at 5 weeks of gestation in humans as CD34^+^ cells in the ventral endothelium of the aorta (Easterbrook et al., 2019; Tavian et al., 1996). HSCs emerge during mid-gestation from a subset of hemogenic arterial endothelial cells (i.e. hemogenic endothelium, HE) via endothelial-to-hematopoietic transition (EHT) (Bertrand and Traver, 2009; Boisset et al., 2015; McGrath et al., 2015; Medvinsky et al., 2011; Mikkola and Orkin, 2006; North et al., 1999; Oberlin et al., 2002; Yokomizo et al., 2001). Newly specified hematopoietic cells are visible as c-Kit^+^ (Kit^+^) cells that bud into the vascular lumen (mainly in the dorsal aorta and umbilical and vitelline arteries) in vertebrate embryos, forming intra-aortic cell clusters (IACs) (Boisset et al., 2015; Jaffredo et al., 1998; Medvinsky et al., 2011). These IACs harbor HSC precursors (i.e. pre-HSCs) (Medvinsky et al., 2011; North et al., 1999; Yokomizo et al., 2001). Newly specified HSCs and pre-HSCs migrate to the fetal liver (FL) (Ema and Nakauchi, 2000; Kumaravelu et al., 2002), where they are mostly found by E12.5 and where they are believed to expand and mature during ontogeny (Ema and Nakauchi, 2000; Ganuza et al., 2022, 2020; Murayama et al., 2006), although new non-invasive approaches, including our own studies, have recently highlighted only limited expansion of cells that contribute to life-long hematopoiesis in the FL (Ganuza et al., 2017, 2022, 2020; Henninger et al., 2017). HSCs eventually migrate to the fetal and neonatal bone marrow (BM) (Bertrand and Traver, 2009; Ganuza et al., 2022; Hall et al., 2022; Medvinsky et al., 2011; Mikkola and Orkin, 2006), where they continue to expand and establish the adult HSC pool before becoming largely quiescent between days 21 and 27 post-birth (P21-P27) (Ganuza et al., 2020). HSCs largely reside in the BM in the postnatal mouse and divide to maintain hematopoietic homeostasis (Ganuza et al., 2020).

Although EHT and transitions between HSC precursors, such as pro-HSCs [VE-cadherin (cadherin 5)^+^CD41 (ITGA2B)^+/lo^CD43 (SPN)^−^CD45 (PTPRC)^−^], Type I pre-HSCs (VE-cadherin^+^CD41^+/lo^CD43^+^CD45^−^c-Kit^+^) or Type II pre-HSCs (VE-cadherin^+^CD45^+^c-Kit^+^), are key steps in HSC development (Chen et al., 2009; Liakhovitskaia et al., 2014; Rybtsov et al., 2014, 2011; Taoudi et al., 2008), little is known about the molecular regulators governing this cellular process. Precise regulation of *Runx1*, its co-factor *Cbfb*, *Gata2*, *Notch1* and *Sox17* are essential during EHT (Chen et al., 2009; Clarke et al., 2013; Corada et al., 2013; Kissa and Herbomel, 2010; Kumano et al., 2003; Lizama et al., 2015; Niki et al., 1997; Okuda et al., 1996; Wang et al., 1996a,b). Similarly, *Gfi1* and *Gfi1b* (transcriptional targets of *Runx1*) and *Hes1* and *Hes5* (*Notch1* transcriptional targets) are required to repress the endothelial program in HE (Guiu et al., 2013; Thambyrajah et al., 2016). *Runx1* is considered a master regulator of EHT (Medvinsky et al., 2011; Ottersbach, 2019) since *Runx1^−/−^* mice lack IACs (North et al., 1999; Yokomizo et al., 2001), morpholinos targeting *Runx1* in zebrafish block EHT (Kissa and Herbomel, 2010) and *Runx1^−/−^* hemogenic endothelium does not yield hematopoietic cells *in vitro* (Lancrin et al., 2009). However, despite recent progress, much remains unknown regarding the molecular regulation of EHT. Insights into these processes have come recently from single-cell transcriptomics (Baron et al., 2018; Canu et al., 2020; Gao et al., 2020; Lummertz da Rocha et al., 2022; Oatley et al., 2020; Vink et al., 2020; Yvernogeau et al., 2020; Zhou et al., 2016; Zhu et al., 2020), such as a description of the transcriptome of the first functional HSCs (Vink et al., 2020), identifying a role for the ligand–receptor couple ADM-RAMP2 and SVEP1 in HSC emergence (Yvernogeau et al., 2020), and uncovering of transcriptional networks (e.g. *Stat3*, *Ybx1* and *App*) governing hematopoiesis (Lummertz da Rocha et al., 2022).

Here, we present evidence that the orphan nuclear receptors NR4A1 and NR4A2 are molecular regulators of EHT. These proteins regulate transcription via interaction with numerous proteins, including other transcription factors, transcriptional co-regulators and kinases (Hamers et al., 2013; Kurakula et al., 2014; Milbrandt, 1988; Rodriguez-Calvo et al., 2017). The nuclear receptor family includes >48 proteins classified as steroid hormone receptors that heterodimerize with retinoid X receptors and orphan nuclear receptors (Kurakula et al., 2014). The NR4A subfamily of orphan nuclear receptors includes NR4A1 (also known as NUR77, TR3, NGFI-B), NR4A2 (also known as NURR1) and NR4A3 (also known as NOR-1) (Kurakula et al., 2014; Rodriguez-Calvo et al., 2017). Nuclear receptors typically contain three structural domains: a C-terminal ligand-binding domain (containing 12 alpha-helices), a central DNA-binding domain (enclosing a double zinc finger) and an N-terminal domain (Hamers et al., 2013; Kurakula et al., 2014). The NR4A ligand-binding domain is filled with amino acid side chains that maintain an active conformation, likely why no ligands have been reported for these receptors (Hamers et al., 2013; Kurakula et al., 2014; Rodriguez-Calvo et al., 2017). Rather, regulation of NR4A receptor activity relies on transcriptional mechanisms, post-translational modifications or protein–protein interactions (Hamers et al., 2013; Kurakula et al., 2014; Rodriguez-Calvo et al., 2017). NR4A receptors have been implicated in many cellular processes, including adaptive and innate immunity, inflammation, cell proliferation, differentiation, cell metabolism and neurological functions (Avagyan et al., 2021; Hamers et al., 2013; Kurakula et al., 2014; Rodriguez-Calvo et al., 2017). They act as tumor suppressors of myeloid leukemogenesis. Their absence induces rapid lethal acute myeloid leukemia (AML) in mice (Mullican et al., 2007). However, prior to this study, they have not been implicated in the ontogeny of the hematopoietic system.

Here, we report that *Nr4a1* and *Nr4a2* play a crucial role during EHT, as loss of these genes compromises IAC formation, the frequency of embryonic HSC precursor populations and the emergence of transplantable HSCs during mid-gestation in mice. This effect is at least in part mediated by the inability of HSC precursors to efficiently downregulate Notch signaling to allow HSC maturation. Thus, the NR4A family of orphan nuclear receptors constitutes a previously unappreciated group of EHT regulators.

## RESULTS

### Nr4a gene expression is upregulated during EHT

**Fig. 1.**
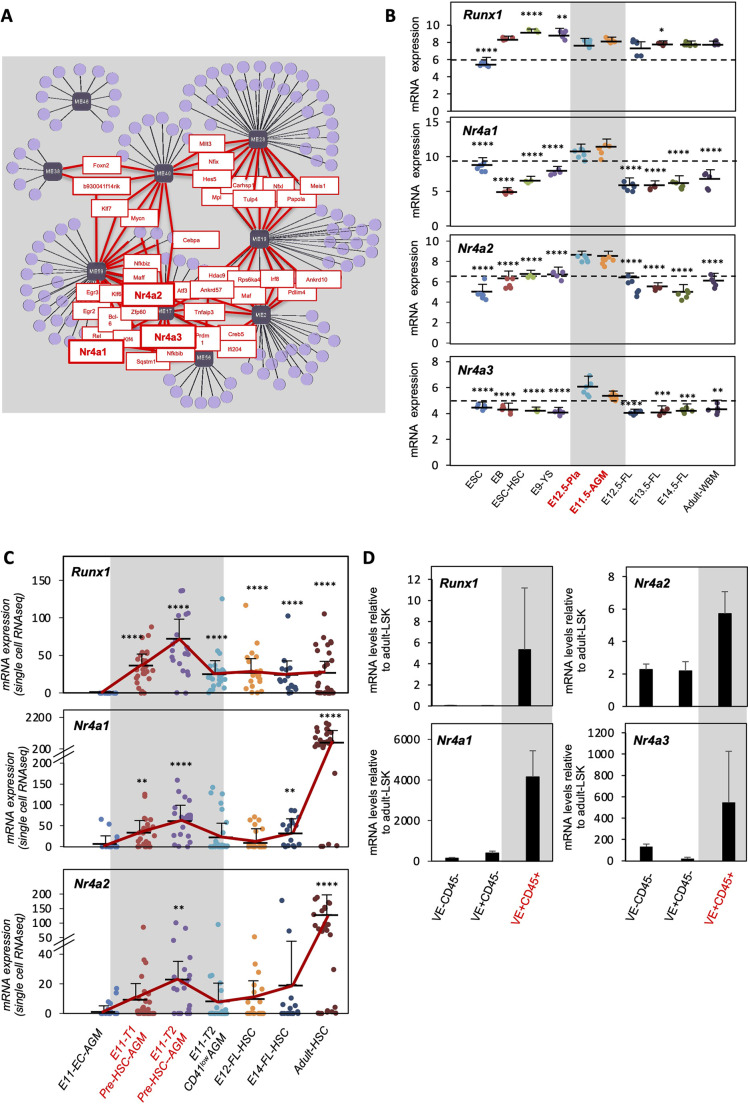
**Nr4a genes are upregulated in the hemogenic endothelium during EHT.** (A) Network schematic of the CLR-derived predictions at the 0.05 FDR for all HSC-specifying modules. Data taken from the StemSite portal (http://daleystem.hms.harvard.edu/; McKinney-Freeman et al., 2012). Predicted transcriptional regulators are highlighted in red. Gene modules shown in gray squares. Names of ‘Hub’ genes are indicated. (B) Expression levels of *Runx1*, *Nr4a1*, *Nr4a2* and *Nr4a3* mRNA extracted from the StemSite portal (McKinney-Freeman et al., 2012). E9-YS (E9-CD41^+^c-Kit^+^CD34^+^ YS cells), E11.5-AGM (E11.5 VE-cadherin^+^CD45^+^ AGM cells), E12.5-Pla (E12.5-CD45^+^c-Kit^+^CD34^med^ placenta cells), E12.5-FL (E12.5 Lin^−^Sca-1^+^c-Kit^+^VE-cadherin^+^Mac-1^low^ FL cells), E13.5-FL (E13.5 Lin^−^Sca-1^+^c-Kit^+^CD150^+^CD48^−^ FL cells), E14.5-FL (E14.5 Lin^−^Sca-1^+^c-Kit^+^CD150^+^CD48^−^ FL cells), adult WBM (Lineage^−^Sca-1^+^c-Kit^+^CD150^+^CD48^−^ adult BM cells), ESC (embryonic stem cells), EB (CD41^+^c-Kit^+^ cells isolated from day six mouse embryoid bodies) and ESC-HSC (CD41^bright^CD45^−^CD34^−^cells) are shown (McKinney-Freeman et al., 2012). (C) Single-cell RNA-sequencing expression analysis of *Runx1*, *Nr4a1* and *Nr4a2* mRNA. Data extracted from figure S3 of Zhou et al. (2016): E11-EC-AGM (E11-CD31^+^VE-cadherin^+^CD41^−^CD43^−^CD45^−^Ter119^−^AGM cells); E11-T1 pre-HSC-AGM (E11 CD31^+^CD45^−^CD41^low^c-Kit^+^CD201^high^ AGM cells); E11-T2 pre-HSC-AGM (CD31^+^CD45^+^c-Kit^+^CD201^high^ cells); E11-T2 CD41^low^CD201^−^ AGM (E11 CD31^+^CD45^+^CD41^low^CD201^−^ AGM cells); E12-FL-HSCs (E12-Lin^−^Sca-1^+^Mac-1^low^CD201^+^ FL cells); E14-FL-HSCs (E14-CD45^+^CD150^+^CD48^−^CD201^+^ FL cells) and adult HSCs (CD150^+^CD48^−^ Lineage^−^Sca-1^+^c-Kit^+^ cells). (D) Quantitative RT-PCR of *Runx1*, *Nr4a1*, *Nr4a2* and *Nr4a3* gene expression in VE-cadherin^−^CD45^−^, VE-cadherin^+^CD45^−^ and VE-cadherin^+^CD45^+^ cells isolated from E11.5 AGMs in two independent experiments by pooling ∼50 AGMs/experiment. Flow cytometry gating strategies are shown in Fig. S1D. In B-D, gray boxes and red letters highlight hemogenic endothelium-related populations. Means and standard deviations are indicated. ****P<0.0001, ***P<0.001, **P<0.01, *P<0.05 (Kruskal–Wallis tests). *n*=2-30. In B,C, each dot represents a different biological sample. In B, statistical differences were tested for each population compared to E11.5 AGM. In C, statistical differences were tested for each population compared to E11 EC-AGM. Source data are provided in Table S1. CD150, SLAMF1; CD201, marker for EPCR (PROCR); Mac-1, ITGAM; Sca-1, LY6A; Ter119, LY76.

We previously examined transcriptional changes across hematopoietic development and identified modules of genes specifically expressed during selected windows of HSC ontogeny (McKinney-Freeman et al., 2012). The context likelihood of relatedness (CLR) algorithm allowed us to identify putative transcriptional regulators (TRs) of each of these gene modules. These analyses implicated the Nr4a family of orphan nuclear receptors (i.e. *Nr4a1*, *Nr4a2* and *Nr4a3*) as putative regulators of multiple stages of ongoing HSC specification (Fig. 1A) (McKinney-Freeman et al., 2012). Moreover, examination of publicly available expression databases (McKinney-Freeman et al., 2012; Zhou et al., 2016) on bulk and single cells derived from mouse embryos further revealed upregulation of *Nr4a1*, *Nr4a2* and *Nr4a3* expression in HSC precursors, similar to *Runx1*, relative to endothelial cells at E11 (Fig. 1B,C). These data show that *Nr4a* genes are upregulated in definitive hematopoietic cells, particularly in pre-HSCs type I and type II. To verify these data, we isolated by fluorescence activated cell sorting (FACS) VE-cadherin^+^CD45^–^ endothelial cells, VE-cadherin^+^CD45^+^ (which are known to contain pre-HSC-type II precursors, as previously described by the Medvinsky laboratory; Rybtsov et al., 2011; Taoudi et al., 2008) and VE-cadherin^–^CD45^–^ cells from E11.5 aorta-gonads-mesonephros (AGMs) and examined by real-time quantitative reverse transcription polymerase chain reaction (qRT-PCR) the expression of *Runx1*, *Nr4a1* and *Nr4a2* (Fig. 1D, Fig. S1A). As expected (North et al., 1999, 2002; Nottingham et al., 2007), *Runx1* was upregulated in VE-cadherin^+^CD45^+^ cells (Fig. 1D). The expression of *Nr4a1*, *Nr4a2* and *Nr4a3* was also upregulated in VE-cadherin^+^CD45^+^ cells (Fig. 1D), confirming that these genes are specifically upregulated in definitive hematopoietic cells.

### HSC specification is sensitive to Nr4a gene dosage

**Fig. 2.**
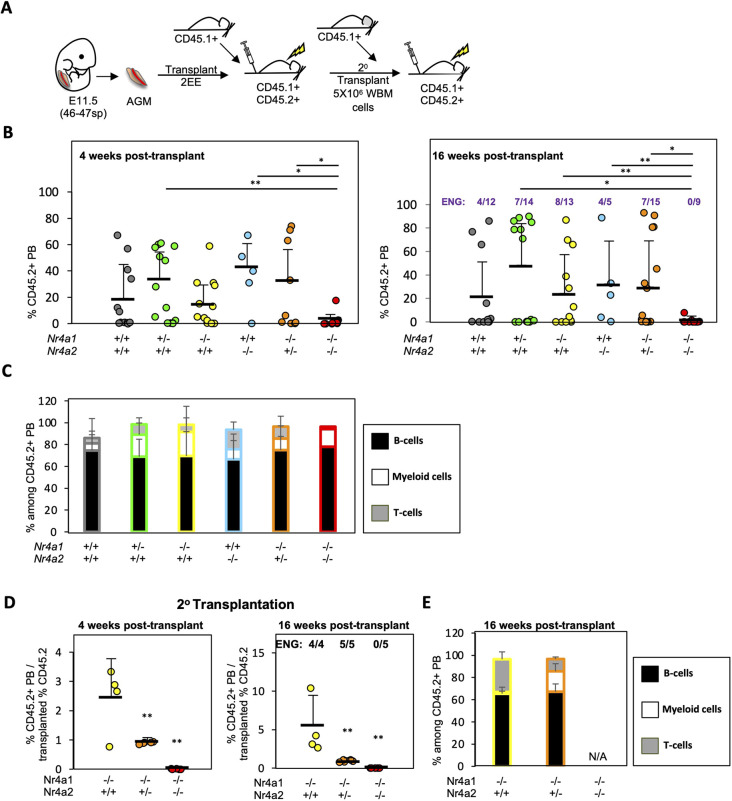
**HSC specification is sensitive to *Nr4a1* and *Nr4a2* gene dosage.** (A) Experimental schematic. AGMs were dissected from E11.5 CD45.2^+^ *Nr4a1^+/+^*, *Nr4a1^+/^*^−^, *Nr4a1*^−*/*−^, *Nr4a2*^−*/*−^, *Nr4a1*^−*/*−^*Nr4a2^+/^*^−^ and *Nr4a1*^−*/*−^*Nr4a2*^−*/*−^ (46-47 sp) concepti, dissociated and transplanted at 2 EE/recipient into lethally irradiated CD45.1^+^CD45.2^+^ mice along with 2×10^5^ CD45.1^+^ WBM cells. After 16 weeks, 5×10^6^ WBM cells isolated from engrafted primary recipients of *Nr4a2*^−*/*−^, *Nr4a1*^−*/*−^*Nr4a2^+/^*^−^ and *Nr4a1*^−*/*−^*Nr4a2*^−*/*−^ AGM-derived cells were transplanted into lethally irradiated CD45.1^+^/CD45.2^+^ congenic secondary recipients. (B) Percentage of CD45.2^+^ PB of primary recipients is shown at 4 (left) and 16 (right) weeks post-transplant. *Nr4a1^+/+^* (*n*=13 recipients), *Nr4a1^+/^*^−^ (*n*=14 recipients), *Nr4a1*^−*/*−^ (*n*=13 recipients), *Nr4a2*^−*/*−^ (*n*=5 recipients), *Nr4a1*^−*/*−^*Nr4a2^+/^*^−^ (*n*=9 recipients) and *Nr4a1*^−*/*−^*Nr4a2*^−*/*−^ (*n*=9 recipients). Data are pooled from ten independent transplants. Each dot represents an independent recipient. The fraction of mice positively engrafted (ENG: defined as %CD45.2^+^ >2% and showing multilineage engraftment) is shown at the top of the graph (16 weeks post-transplant). **P<0.01, *P<0.05 (Wilcoxon rank sum test). (C) Percentage of B cells, T cells and myeloid cells within CD45.2^+^ PB of recipients engrafted with >1% of CD45.2^+^ PB at 16 weeks post-transplant. (D) The ratio of reconstituted %CD45.2^+^ PB in secondary recipients and to %CD45.2^+^ transplanted BM is depicted at 4 (left) and 16 (right) weeks post-transplant. For each genotype, BM from a single primary recipient was transplanted. Each circle represents an independent secondary recipient. The fraction of mice positively engrafted (ENG: defined as %CD45.2^+^ >2% and multilineage engraftment) is shown at the top of the 16 week panel. **P<0.01 (Wilcoxon rank sum test). (E) Percentage of B cells, T cells and myeloid cells within CD45.2^+^ PB of recipients engrafted with >1% of CD45.2^+^ PB at 16 weeks post-transplant. In B-E, mean and standard deviation are shown. Source data are provided in Table S1. N/A, not applicable.

To functionally explore whether Nr4a genes are necessary for HSC specification, we took advantage of *Nr4a1^−/−^* and *Nr4a2^−/−^* mice (Lee et al., 1995; Pan et al., 2008; Zetterstrom et al., 1997). *Nr4a2^−/−^* mice die perinatally, whereas *Nr4a1^−/−^* and *Nr4a2^+/−^* mice are viable, fertile and do not display any gross physical abnormalities. Although *Nr4a1^−/−^Nr4a2^−/−^* embryos were not observed at Mendelian ratios (Fig. S2A), all the *Nr4a1^−/−^Nr4a2^−/−^* embryos we obtained had a visible heartbeat and their morphology (Fig. S2B) and AGM cellularity (Fig. S2C) were normal at E12.5, suggesting that these embryos have no overt defects at least until after E12.5. To assess whether HSC specification is affected in the absence of *Nr4a1* and *Nr4a2*, we isolated AGMs from CD45.2^+^ *Nr4a^+/+^*, *Nr4a1^+/−^*, *Nr4a1^−/−^*, *Nr4a2^−/−^*, *Nr4a1^−/−^Nr4a2^+/−^* and *Nr4a1^−/−^Nr4a2^−/−^* concepti at E11.5 and transplanted into lethally irradiated CD45.1^+^CD45.2^+^ congenic recipients at a dose of two AGMs (or embryo-equivalents, EE) per recipient together with 2×10^5^ CD45.1^+^ whole BM (WBM) cells (Fig. 2A).

Remarkably, 0/9 recipients of *Nr4a1^−/−^Nr4a2^−/−^* AGM-derived cells displayed tri-lineage (i.e. myeloid, B cell and T cell) CD45.2^+^ peripheral blood (PB) engraftment at 4 and 16 weeks post-transplant (Fig. 2B). Only one recipient displayed donor-derived PB, but this was restricted to the myeloid and B cell lineage (Fig. 2C, Fig. S1B). In contrast, numerous animals transplanted with *Nr4a^+/+^* (4/12), *Nr4a1^+/−^* (7/14), *Nr4a1^−/−^* (8/13), *Nr4a2^−/−^* (4/5) or *Nr4a1^−/−^Nr4a2^+/−^* (7/15) displayed tri-lineage CD45.2^+^ PB engraftment (Fig. 2B,C).

Next, to assess the self-renewal potential of Nr4a-deficient hematopoietic stem and progenitor cells (HSPCs), WBM was isolated from primary recipients stably engrafted with *Nr4a1^−/−^Nr4a2^+/+^*, *Nr4a1^−/−^Nr4a2*^+*/−*^ or *Nr4a1^−/−^Nr4a2^−/−^* AGM-derived cells and transplanted into lethally irradiated secondary CD45.1^+^CD45.2^+^ recipients (Fig. 2A). Although all secondary recipients of *Nr4a1^+/+^Nr4a2^−/−^* and *Nr4a1^−/−^Nr4a2*^+/*−*^ AGM-derived cells displayed CD45.2^+^ PB engraftment, *Nr4a1^−/−^Nr4a2^−/−^* AGM-derived cells (transplanted from the sole partially engrafted primary recipient) failed to contribute to the PB of secondary recipients (Fig. 2D). Furthermore, *Nr4a1^−/−^Nr4a2^+/−^* AGM-derived cells displayed significantly lower secondary engraftment of the PB than *Nr4a1^+/+^Nr4a2^−/−^* AGM-derived cells. These data reveal that both HSC specification and the self-renewal potential of newly specified HSCs are sensitive to Nr4a gene dosage. Interestingly, *Nr4a1^−/−^Nr4a2^+/+^-*derived HSPCs were devoid of secondary myeloid potential (Fig. 2D,E). This defect in self-renewal may indicate that the emerged *Nr4a1^−/−^Nr4a2^+/−^* HSCs are not fully functional and the exposure to a wild-type adult BM microenvironment is not able to rewire/rescue their developmental defect. Alternatively, *Nr4a1* and *Nr4a2* genes may be required in adult hematopoiesis to maintain HSC function. Further investigations employing conditional knockout of Nr4a alleles would be required to formally address this.

### EHT is sensitive to Nr4a gene dosage

**Fig. 3.**
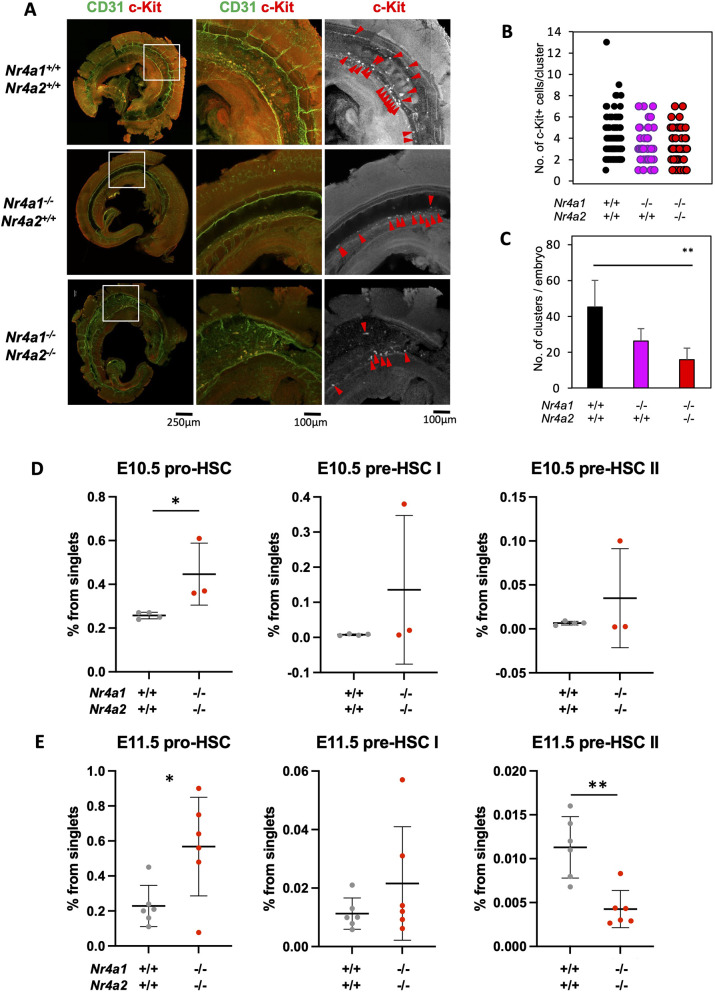
**HSC specification depends on *Nr4a1* and *Nr4a2* gene dosage.** (A) Representative confocal images of c-Kit^+^ IACs in E10.5 *Nr4a1^+/+^* (*n*=4), *Nr4a1*^−*/*−^ (*n*=5) and *Nr4a1*^−*/*−^*Nr4a2*^−*/*−^ (*n*=6) embryos. Embryos were examined from three independent experiments. For each representative embryo, the area containing the highest concentration of c-Kit^+^ clusters is highlighted (boxed area) and shown at higher magnification in panels to the right. Anti-CD31 is shown in green and c-Kit in red. Red arrowheads indicate c-Kit+ IACs. Scale bars: 250 µm (left); 100 µm (middle and right). (B) Quantification of the number of c-Kit^+^ cells/IAC in the dorsal aorta. Each dot represents a single cluster. (C) Quantification of total IACs/embryo in the dorsal aorta. In B,C, graphs show pooled results from all embryos analyzed. Mean and standard deviation are shown. **P<0.01 (Wilcoxon rank sum test). (D,E) Frequency of pro-HSCs, pre-HSC type I and pre-HSC type II in *Nr4a1* and *Nr4a2* E10 (D) and E11.5 (E) mutant embryos. Graphs show pooled results from 12 independent experiments. Mean and standard deviation are shown. Statistical differences highlight the difference among *Nr4a1*^+/+^*Nr4a2*^+/+^ embryos and the other groups shown. **P<0.01, *P<0.05 (unpaired, two-sample *t*-tests and Mann–Whitney tests). Source data are provided as a source data file in Table S1.

Upregulation of Nr4a gene expression in VE-cadherin^+^CD45^+^ HE relative to the endothelium in the E11.5 AGM (Fig. 1) and the lack of engraftment potential in *Nr4a1^−/−^Nr4a2^−/−^* E11.5 embryos (Fig. 2) suggest that Nr4a genes may play a role in EHT. c-Kit^+^ IACs emerge from the hemogenic endothelium. Any perturbation of EHT should affect the number and/or size of IACs. Hence, to explore the role of Nr4a genes in EHT, we evaluated HSC specification at E10.5, which constitutes the peak of IAC numbers in the embryo (Medvinsky et al., 2011; Yokomizo and Dzierzak, 2010; Yokomizo et al., 2011). We examined E10.5 *Nr4a1^+/+^Nr4a2^+/+^*, *Nr4a1^−/−^Nr4a2^+/+^*, *Nr4a1^−/−^Nr4a2^−/−^* embryos for c-Kit^+^ IACs using confocal microscopy (Fig. 3A). CD31 (PECAM1) expression was used to identify endothelium. As mentioned, c-Kit^+^ IACs are formed by HSC precursors budding from the hemogenic endothelium into the lumen of the dorsal aorta via EHT. Despite this, we detected no differences in the number of c-Kit^+^ cells per cluster (Fig. 3B), *Nr4a1^−/−^Nr4a2^−/−^* embryos displayed significantly fewer IACs than *Nr4a1^+/+^Nr4a2^+/+^* embryos at E10.5 (Fig. 3A,C).

We next examined the maturation of endothelial precursors into hematopoietic cells by flow cytometry analysis of HSC precursor populations such as pro-HSC (VE-cadherin^+^CD41^+/lo^CD43*^−^*CD45*^−^*), Type I pre-HSC (VE-cadherin^+^CD41^+/lo^CD43^+^CD45*^−^*c-Kit^+^) and Type II pre-HSC (VE-cadherin^+^CD45^+^c-Kit^+^) HSC precursors (Chen et al., 2009; Liakhovitskaia et al., 2014; Rybtsov et al., 2014, 2011; Taoudi et al., 2008) (Fig. 3D,E, Fig. S1C). Interestingly, we observed a blockage in HSC maturation with Nr4a gene dosage reduction. Particularly, the number of pro-HSCs was significantly increased in *Nr4a1^−/−^Nr4a2^−/−^* E10.5 and E11.5 embryos (Fig. 3D,E). Although differences were not statistically significant, Pre-HSCs-type I progenitors also accumulated in *Nr4a1^−/−^Nr4a2^−/−^* E10.5 and E11.5 embryos. Interestingly, we observed a significant decrease in the numbers of more mature pre-HSCs-type II in E11.5 *Nr4a1^−/−^Nr4a2^−/−^* embryos, suggesting an impaired HSC specification process in the AGM (Fig. 3D,E).

Overall, our data indicate that loss of *Nr4a1* and *Nr4a2* genes perturbs EHT in the AGM.

### HSC specification is rescued by *ex vivo* culture of E10.5 Nr4a-deficient embryos

**Fig. 4.**
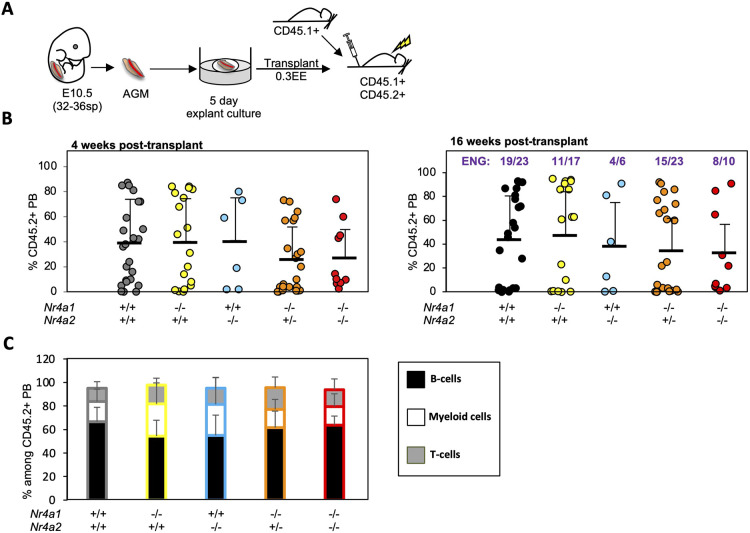
***Ex vivo* cultured E10.5 Nr4a-deficient AGMs display robust HSC activity.** (A) Experimental schematic. AGMs were dissected from E10.5 (32-36 sp) concepti and cultured as explants at the air–liquid interface for 5 days. Explants were then harvested, dissociated and transplanted at 0.3 EE/recipient. (B) CD45.2^+^ PB of primary recipients at 4 (left) and 16 (right) weeks post-transplant. *Nr4a1^+/+^* (*n*=24 recipients), *Nr4a1*^−*/*−^ (*n*=18 recipients), *Nr4a2*^−*/*−^ (*n*=6 recipients), *Nr4a1*^−*/*−^*Nr4a2^+/^*^−^ (*n*=23 recipients) and *Nr4a1*^−*/*−^*Nr4a2*^−*/*−^ (*n*=10 recipients). Data were pooled from six independent experiments. Each dot represents an independent recipient. The fraction of mice positively engrafted (ENG: defined as %CD45.2^+^ >2% and multilineage engraftment) is shown at the top of the graph (16 weeks post-transplant). (C) Percentage of B cells, T cells and myeloid cells within CD45.2^+^ PB of recipients engrafted with >1% of CD45.2^+^ PB at 16 weeks post-transplant. Means and standard deviations are shown. Source data are provided in Table S1.

E10.5 embryos provide <1 transplantable HSC per embryo (Medvinsky et al., 2011). However, *ex vivo* culture of E10.5 AGM explants in the presence of supraphysiological levels of SCF (KITL), IL3 and FLT3L, allows for the continued specification, maturation and amplification of transplantable HSCs (Ganuza et al., 2018, 2017; Taoudi et al., 2008). Thus, to further test the role of Nr4a genes during EHT, we examined whether HSC specification could be coaxed from E10.5 embryos *ex vivo*: E10.5 AGMs from CD45.2^+^ *Nr4a^+/+^*, *Nr4a1^−/−^*, *Nr4a2^−/−^*, *Nr4a1^−/−^Nr4a2^+/−^* and *Nr4a1^−/−^Nr4a2^−/−^* embryos were cultured as whole explants for 5 days at the air/liquid interface in culture media supplemented with SCF, FLT3L and IL3, as previously described (Fig. 4) (Ganuza et al., 2018, 2017; Taoudi et al., 2008). Only embryos with 32-36 somite pairs (sp) were used in these experiments for consistency. After 5 days of culture, explants were dissociated and transplanted at 0.3 EE/recipient into lethally irradiated CD45.1^+^CD45.2^+^ mice along with 2×10^5^ CD45.1^+^ WBM cells (Fig. 4A). Surprisingly, *Nr4a1^−/−^Nr4a2^−/−^* explants contributed to the PB of 8/10 recipients up to 16 weeks post-transplant (Fig. 4B,C), albeit at lower levels of engraftment than mice transplanted with *Nr4a^+/+^* explants. Indeed, there appears to be a marginal direct correlation between Nr4a gene dosage and engraftment potential: the lower the Nr4a gene dosage, the lower the engraftment potential of CD45.2^+^ donor embryos, although these differences were not statistically significant (Fig. 4B,C). These data reveal that E10.5 *Nr4a1^−/−^Nr4a2^−/−^* embryos retain their potential for HSC specification or expansion under exogenous exposure to SCF, FLT3L and IL3.

In summary, the cellular and functional defects observed in HSC hematopoietic progenitors in *Nr4a1^−/−^Nr4a2^−/−^* embryos strongly support a role for the Nr4a gene family at the onset of definitive hematopoiesis.

### *Nr4a1^−/−^Nr4a2^−/−^* IACs fail to downregulate Notch signaling

**Fig. 5.**
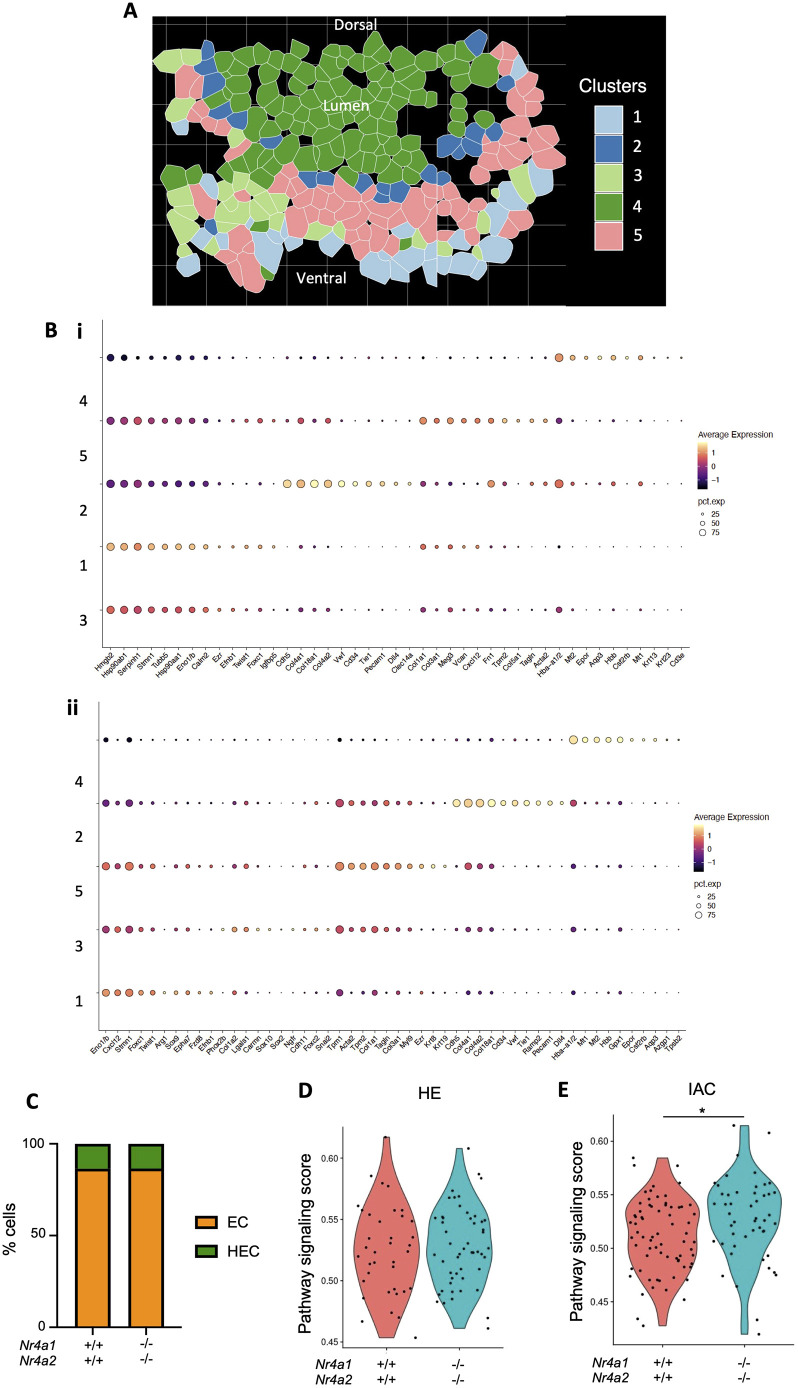
**Spatial transcriptomics (CosMx) reveals an aberrant persistence of Notch signaling during EHT in *Nr4a1***^−*/*−^***Nr4a2***^−*/*−^ **embryos.** (A) Representative image of the ventral aspect of the dorsal aorta area that was selected in each section for CosMx analysis, showing the different cell identity clusters obtained. Twelve *Nr4a1^+/+^Nr4a2^+/+^* sections and seven *Nr4a1*^−*/*−^*Nr4a2*^−*/*−^ sections that were intact after processing were used for downstream analysis. Sections of each embryo were combined and unsupervised clustering analysis was performed independently for each embryo. (B) Dotplots showing the top ten most highly expressed genes for each cluster in *Nr4a1^+/+^Nr4a2^+/+^* (i) and *Nr4a1*^−*/*−^*Nr4a2*^−*/*−^ (ii) embryos. (C) Comparison of the percentage of HECs (Runx1^+^ ECs) and arterial endothelial cells (Runx1^–^ ECs) between *Nr4a1^+/+^Nr4a2^+/+^* and *Nr4a1*^−*/*−^*Nr4a2*^−*/*−^ embryos. (D,E) Violin plots showing the Notch pathway signaling score in HE (D; *P*=0.7138) and IACs (E; *P=0.02173) between *Nr4a1^+/+^Nr4a2^+/+^* and *Nr4a1*^−*/*−^*Nr4a2*^−*/*−^ embryos. A two-tailed, unpaired *t*-test was used. *P<0.05. Source data are provided in Table S1.

To unveil the molecular mechanism by which NR4A1 and NR4A2 mediate HSC emergence, we performed a high-plex *in situ* analysis using a CosMx^TM^ spatial molecular imager (SMI) on sections from one *Nr4a^+/+^Nr4a2^+/+^* and one *Nr4a1^−/−^Nr4a2^−/−^* E10 embryo. Each slide contained sections spanning from the anterior to the posterior AGM region (Fig. S3A) and were hybridized with a panel of 950 pre-set probes (provided by NanoString) and 50 custom probes (Table S2). Following cell segmentation (Fig. S3A) and data curation, we selected the ventral aspect of the dorsal aorta and adjacent niche cells as our area of interest for each section (Fig. 5A). For each embryo, unsupervised clustering analysis of combined sections was performed independently (Fig. 5A,B). Based on cellular location and most highly expressed genes (*Cdh5*, *Pecam1*, *Vwf*, *Dll4*, etc.), we could unequivocally identify cluster 2 as the cluster containing endothelial cells (ECs) in both *Nr4a^+/+^Nr4a2^+/+^* and *Nr4a1^−/−^Nr4a2^−/−^* embryos (Fig. 5A,B). We also identified hematopoietic cells (HCs) in cluster 4 as cells with high levels of hematopoietic genes, such as *Hbb*, *Csf2rb*, *Epor* and *Cd3e*. Most cells in this cluster are found in the lumen of the dorsal aorta, and several of the genes in this cluster are typically expressed in primitive hematopoietic cells (Wittamer and Bertrand, 2020). Cluster 5 has a more pronounced smooth muscle signature, with high expression of genes such as *Tgln* (*Tagln*) and *Acta2*, and the cells are proximal to the endothelium (Gonzalez Galofre et al., 2024). Cluster 1 seems to have a sclerotome signature, based on the high expression of genes such as *Foxc1* and *Twist1* (Della Gaspera et al., 2019; Kume et al., 2001)*.* Cluster 3, however, differs between *Nr4a1^+/+^Nr4a2^+/+^* and *Nr4a1^−/−^Nr4a2^−/−^* embryos. It appears to have a neural crest signature, with the expression of *Sox10*, *Phox2b* and *Ngfr* (Kapeni et al., 2022; Pattyn et al., 1999), but exhibits an unclear signature in the *Nr4a1*^+/+^*Nr4a2*^+/+^ embryos (Fig. 5A,B).

We compared the percentage of *Runx1*-expressing ECs (hemogenic endothelial cells, HECs) between *Nr4a^+/+^Nr4a2^+/+^* and *Nr4a1^−/−^Nr4a2^−/−^* and found no differences (Fig. 5C), suggesting that the emergence of *Runx1*-expressing HECs is not altered in *Nr4a1^−/−^Nr4a2^−/−^* embryos.

Since EHT requires an initial upregulation of Notch signaling followed by its subsequent downregulation for successful completion (Ditadi et al., 2015; Gama-Norton et al., 2015; Kobayashi et al., 2014; Lizama et al., 2015; Porcheri et al., 2020; Richard et al., 2013; Sacilotto et al., 2013; Souilhol et al., 2016; Thambyrajah and Bigas, 2022; You et al., 2005), we compared Notch pathway activation between *Nr4a^+/+^Nr4a2^+/+^* and *Nr4a1^−/−^Nr4a2^−/−^* embryos. Interestingly, we found no differences in Notch pathway activation between genotypes in HECs (Fig. 5D), suggesting that the hematopoietic defects observed in *Nr4a1^−/−^Nr4a2^−/−^* embryos occur after the emergence of *Runx1*-expressing HECs.

We next evaluated Notch signaling activity in IACs. Because IACs are composed of cells transitioning from an endothelial to a hematopoietic identity, we identified IACs as cells co-expressing *Cdh5* and *Kit* within both cluster 2 (ECs) and cluster 4 (HCs). Remarkably, we found that Notch signaling activity was significantly increased in *Nr4a1^−/−^Nr4a2^−/−^* embryos (Fig. 5E). These data support the hypothesis that *Nr4a1^−/−^Nr4a2^−/−^* IACs are unable to effectively transition through EHT, preventing their maturation into HSCs.

Since sterile pro-inflammatory pathways have been implicated in the regulation of EHT in fish and mice (Espin-Palazon et al., 2014; He et al., 2015; Li et al., 2014; Ottersbach, 2019) and considering that all three NR4A proteins are early-response genes expression of which is rapidly activated by NF-κB-mediated pro-inflammatory insults (Harant and Lindley, 2004; Hong et al., 2004; Rodriguez-Calvo et al., 2017; Saijo et al., 2009), we also assessed changes in the NF-κB signaling pathway in *Nr4a1^−/−^Nr4a2^−/−^* embryos. We failed to detect significant changes in this pathway (Fig. S3C,D), indicating that NR4As do not modulate EHT via this sterile-pro-inflammatory pathway.

## DISCUSSION

Illuminating the molecular mechanisms that regulate HSC emergence during ontogeny is paramount to optimizing protocols to induce HSC specification *in vitro*. Here, we present evidence that NR4A orphan nuclear receptors are required for efficient EHT during HSC specification. Reduced *Nr4a1* and *Nr4a2* gene dosage results in a loss of IACs, a blockage in HSC specification and compromised HSC self-renewing ability in E11.5 embryos. The loss of detectable transplantable HSCs was partially rescued when supraphysiological levels of SCF, IL3 and FLT3L were administered *in vitro* to E10.5 AGM explants.

During EHT, *Nr4a1*, *Nr4a2* and *Nr4a3* transcripts are upregulated in E11.5 type II pre-HSCs (VE-cadherin^+^CD45^+^) and CD31^+^CD45^−^CD41^low^c-Kit^+^CD201^high^ pre-HSCs, which express higher Nr4a transcript levels than E11.5 endothelium (including VE-cadherin^+^CD45^–^ and CD31^+^VE-cadherin^+^CD41^−^CD43^−^CD45^−^Ter119^−^) (Fig. 1).

Further supporting our expression analysis, we observed the same *Nr4a1* expression pattern in a recent single-cell RNA-sequencing dataset that studied the dynamics of HSC production in the aorta (Baron et al., 2018). In particular, *Nr4a1* was upregulated in hemogenic endothelial cells (defined as CDH5^+^GFI1^+^c-Kit^−^) versus non-HE cells (defined as CDH5^+^GFI1^−^c-Kit*^−^*) at E10 (2.58-fold, *P*=0.0001) (Baron et al., 2018). Moreover, our CLR analysis identified all three NR4As as putative transcriptional regulators of specification of gene modules, i.e. modules of genes expression of which is particularly upregulated in E11-VE-cadherin^+^CD45^+^ type II pre-HSCs (Fig. 1A).

We found that the combined loss of *Nr4a1* and *Nr4a2* precluded the detection of transplantable HSCs in E11.5 embryos (Fig. 2). This could result from defective EHT and/or a problem with recently emerged *Nr4a-*deficient c-Kit^+^ cells maturing into transplantable HSCs. Importantly, *Nr4a1^−/−^Nr4a2^−/−^* embryos showed loss in the numbers of IACs (Fig. 3A,C). As IACs emerge via EHT from hemogenic endothelium, these defects implicate NR4A receptors in the regulation of EHT. Furthermore, the blockage in HSC specification, noted as an accumulation of pro-HSCs and Type I pre-HSCs in *Nr4a1^−/−^Nr4a2^−/−^* embryos and the reduced numbers of more mature Type II pre-HSCs in *Nr4a1^−/−^Nr4a2^−/−^* embryos, strongly indicates a role of Nr4a genes in EHT (Fig. 3).

NR4A nuclear receptors coordinate many cellular processes, including metabolism, DNA repair, cell proliferation and apoptosis (Kurakula et al., 2014; Rodriguez-Calvo et al., 2017). NR4A nuclear receptors contain a nearly identical DNA-binding domain (Hamers et al., 2013; Kurakula et al., 2014; Rodriguez-Calvo et al., 2017). However, their N-terminal AF-1 ligand-independent domain is very divergent and likely confers functional specificity to each family member (Kurakula et al., 2014). NR4As interact with multiple other transcription factors, which modulates their activity. As monomers, they bind the NGFI-B response element in the promoters of genes targets, as NR4A homodimers or NR4A heterodimers they bind Nur-response elements, and as NR4A-RXR (retinoid X receptors) heterodimers they bind to DR-5 sequences (Kurakula et al., 2014; Rodriguez-Calvo et al., 2017). In particular, the NR4A1 interactome has been extensively studied and, notably, NR4A1 was reported to bind NOTCH1 (Jehn et al., 1999).

Importantly, NOTCH1 signaling is essential for HSC development both in mouse and zebrafish embryos (Bertrand et al., 2010b; Hadland et al., 2004; Kumano et al., 2003). In particular, *Notch1^−/−^* embryonic stem cells fail to contribute to the adult hematopoietic system of chimeric mice (Hadland et al., 2004), and *Notch1^−/−^* embryos develop small numbers of colony-forming-units in culture (Hadland et al., 2004; Kumano et al., 2003). Moreover, NOTCH1 regulates *Gata2* (Robert-Moreno et al., 2005, 2008), which controls *Runx1* (Nottingham et al., 2007). *Gata2* haploinsufficiency results in fewer HSCs (Ling et al., 2004), and embryos lacking *Hes1* and *Hes5* (known NOTCH1 transcriptional targets) show sustained *Runx1* expression due to a failure to downregulate *Gata2* expression, leading to larger IACs (Guiu et al., 2013). Importantly, these clusters are non-functional, suggesting that NOTCH1, GATA2 and RUNX1 are required to initiate EHT, but must be downregulated to complete EHT (Guiu et al., 2013; Ottersbach, 2019).

These and other evidences (Ditadi et al., 2015; Gama-Norton et al., 2015; Kobayashi et al., 2014; Lizama et al., 2015; Porcheri et al., 2020; Richard et al., 2013; Sacilotto et al., 2013; Souilhol et al., 2016; Thambyrajah and Bigas, 2022; You et al., 2005) highlight that Notch signaling is exquisitely regulated during EHT, and that EHT can be divided into two phases that we are referring to now as ‘initiation’ and ‘completion’. Our spatial transcriptomics data show that Notch signaling activity is not perturbed in *Nr4a1^−/−^Nr4a2^−/−^ Runx1*-expressing HECs but is upregulated in *Nr4a1^−/−^Nr4a2^−/−^* IACs (Fig. 5D), positioning NR4A proteins as essential regulators of the transition from EHT initiation to completion. The accumulation of pro-HSCs at E10.5 and E11.5 and the reduction of pre-HSC type II at E11.5 further confirmed their role regulating this EHT transition, which likely results in the reduced number of IACs seen in *Nr4a1^−/−^Nr4a2^−/−^* embryos (Fig. 3B).

Additionally, Notch has been reported to regulate arterial endothelial identity (Phng and Gerhardt, 2009) by suppressing COUP-TFII (NR2F2), an orphan receptor that blocks NOTCH signaling. Importantly, IACs only emerge from arterial endothelium (Medvinsky et al., 2011; You et al., 2005) and NR4A1 also can bind and repress COUP-TFII transcriptional activity (Wu et al., 1997). Thus, NR4As could potentially modulate Notch at this level as well.

DNA motif binding analysis suggests that NR4A1 may cooperate with RUNX1 to regulate gene expression in AML Kasumi cells (Duren et al., 2016). Additionally, NR4A2 can directly bind RUNX1 in T cells, although the effect of this binding is unknown (Sekiya et al., 2013). As RUNX1 is required for EHT, any modulation of RUNX1 transcriptional activity via NR4A interaction may impact EHT, which provides another route to be explored in the future on the different ways NR4A proteins may influence EHT.

Intriguingly, *in vitro* culture of E10.5 *Nr41^−/−^Nr4a2^−/−^* AGM explants in conditions that allow for HSC maturation and expansion rescued the engraftment defect of E11.5 *Nr4a1^−/−^Nr4a2^−/−^* AGMs (Fig. 5) (Ganuza et al., 2017; Taoudi et al., 2008). We speculate that the ectopic activation or inhibition of pathways downstream of NR4A proteins (including the inhibition of Notch-related target genes), may bypass their role and induce or resume cell proliferation and/or maturation of E10.5 HSC hemogenic endothelial precursors. Regarding the explant culture conditions that rescued HSC specification defects, while exogenous FLT3L is dispensable for HSC maturation *ex vivo* (Ganuza et al., 2018; Rybtsov et al., 2014), IL3 drives HSC maturation in the AGM between E10.5 and E11.5 (Ganuza et al., 2018; Gordon-Keylock et al., 2013; Robin et al., 2006). Notably, SCF is required for the maturation of E8.5 HSC precursors, E9.5 pro-HSCs and E10.5 type I and type II pre-HSCs (Ganuza et al., 2018, 2017; Rybtsov et al., 2014; Taoudi et al., 2008). Interestingly, SCF is expressed in the dorsal aorta at E9.5-E11.5 (Rybtsov et al., 2014, 2011; Taoudi et al., 2008) and *Scf^−/−^* mice die *in utero* as a result of severe anemia and lack HSCs (Broudy, 1997; Ding et al., 2012). SCF binding induces c-Kit dimerization, kinase activity and signal transduction via many pathways (e.g. PI3-K, JAK/STAT, PLC-γ and MAPK pathways), which control cell proliferation, differentiation, migration and apoptosis (Cardoso et al., 2017; Linnekin, 1999).

In summary, our study reveals a role for the NR4A proteins during EHT and HSC specification. There are currently no robust methods to efficiently expand HSCs *in vitro* or to derive HSCs from other cell sources to model hematologic disease or for cell therapy. Thus, any insight into the molecular regulation of HSC specification may facilitate the optimization of protocols to yield clinically relevant numbers of HSCs for wide transplantation purposes.

## MATERIALS AND METHODS

### Mice

C57BL/6J, C57BL/6.SJL-PtprcaPep3b/BoyJ and B6.129S2-*Nr4a1^tm1Jmi^/*J (*Nr4a1^−/−^*) mice were acquired from The Jackson Laboratory and housed in a pathogen-free facility. The *Nr4a2^−/−^* mouse strain was kindly provided by Orla M. Conneely (Baylor College of Medicine, Houston, TX, USA). *Nr4a1^−/−^* and *Nr4a2^−/−^* were backcrossed with C57BL/6J mice for a minimum of seven generations. All animal experiments were carried out according to procedures approved by the St. Jude Children's Research Hospital Institutional Animal Care and Use Committee and comply with all relevant ethical regulations regarding animal research.

### Genotyping

Polymerase chain reactions (PCR) were performed using Go Taq DNA Polymerase (Promega) to detect *Nr4a2* alleles and HotStarTaq DNA Polymerase (QIAGEN) to detect *Nr4a1* alleles, as indicated by the manufacturers. PCR conditions: (95°C, 2′);[(94°C, 30″; 56°C, 30″; 72°C, 30″)×35]; (72°C, 10′). Primers used were: *Nr4a2* common (5′-GGCACTCCTGTGTCTAGCTGCC-3′), *Nr4a2* mut (5′-CTGCCTTGGGAAAAGCGCCTCC-3′), *Nr4a2* wt (5′-CAGCCCTCACAAGTGCGAACAC-3′). oIMR2060 (*Nr4a1* mut) (5′-CACGAGACTAGTGAGACGTG-3′), oIMR6602 (*Nr4a1* common) (5′-CCACGTCTTCTTCCTCATCC-3′), oIMR6603 (*Nr4a1* wt) (5′-TGAGCAGGGACTGCCATAGT-3′). *Nr4a2* wt PCR band: 300 bp. *Nr4a2* mut PCR band: 200 bp. *Nr4a1* wt PCR band: 180 bp. *Nr4a1* mut PCR band: 350 bp.

Genotypes were determined from a portion of the head of embryos or from tail biopsies from adult mice.

### Embryo isolation and explant culture

Embryos were isolated, stained, tissue-cleared and imaged as previously described (Yokomizo and Dzierzak, 2010; Yokomizo et al., 2012). A portion of the head was used for genotyping. Explant culture conditions were adapted from published protocols (Taoudi et al., 2008). Embryo explants were cultured at the air–liquid interface on 0.65 µm DV Durapore Membrane Filters (Merck Millipore) in Iscove's Modified Dulbecco's Medium-Glutamax (IMDM-Glutamax, Thermo Fisher Scientific), 20% fetal calf serum (FCS) (FB-02, Lot #535905, Omega Scientific), 0.1 mM 2-mercaptoethanol (Thermo Fisher Scientific), and 100 units/ml of Penicillin/Streptomycin (Thermo Fisher Scientific) supplemented with recombinant mouse SCF, recombinant mouse IL3, and recombinant mouse FLT3 ligand (Peprotech; all at 100 ng/ml) for 5 days to allow engraftment from E10.5 tissues. Membrane filters were sterilized and placed on in-house-made ring stands previously located in a well containing 2.25 ml of the above-described media in a non-tissue-culture-treated 6-well-plate (Corning), as previously described (Ganuza et al., 2018). Explants were recovered from filters using sterilized scalpels. Collagenase (0.0012 g/ml, Sigma-Aldrich) in PBS (Thermo Fisher Scientific) supplemented with 10% FCS (Omega Scientific) was used to dissociate cultured explants.

### Transplants

All cells were transplanted by tail vein injection. E10.5 and E11.5 CD45.2^+^ cells were transplanted along with 2×10^5^ CD45.1^+^ C57BL/6.SJL WBM cells into lethally irradiated CD45.2^+^/CD45.1^+^ C57BL/6J congenic recipient mice. Freshly isolated E11.5 embryos dissociated with collagenase were transplanted as 2 EE/recipient. AGM explant cultures from E10.5 embryos were dissociated and transplanted after 5 days of *ex vivo* culture (as described above) as 0.3 EE/recipient. CD45.2^+^/CD45.1^+^ C57BL/6J recipients were treated at 8-12 weeks old with 11 Gy of ionizing radiation in split doses of 5.5 Gy prior to transplant. For secondary transplants, 5×10^6^ WBM cells isolated from primary recipients was transplanted along with 2×10^5^ CD45.1^+^ C57BL/6.SJL WBM cells into lethally irradiated CD45.2^+^/CD45.1^+^ recipients. Engraftment was defined as >2% CD45.2^+^ cells in each lineage (T cells, B cells, and myeloid cells) and >1% CD45.2^+^ total PB.

### PB analysis

PB was collected in heparinized capillary tubes (Fisherbrand) from the retro-orbital plexus, lysed in red blood cell lysis buffer (Sigma-Aldrich) and stained as previously described (Ganuza et al., 2017) with the following antibodies: CD45.1-APC (A20) (110713, BioLegend) or CD45.1-FITC (A20) (561871, BD Biosciences), B220-PECy7 (B220, 60-0452-U025, Tonbo Biosciences), CD8-PECy7 (53-6.7) (60-0081-U100, Tonbo Biosciences), and CD45.2-V500 (104) (562130), B220- PerCPCy5.5 (RA3-6B2) (552771), Gr1-PerCPCy5.5 (RB6-8C5) (552093), Cd11b-PerCPCy5.5 (M1/70) (550993) and CD4-PECy7 (RM4-5) (552775) (BD Biosciences). All antibodies were used at 1:200 dilution. 4′,6-Diamidino-2-phenylindole (DAPI) staining was used to gate live events. Analysis was performed on a LSR Fortessa (BD Biosciences). Cells were collected employing the BD FACSDiva Software (v.8.0.1) (BD Biosciences). The data were analyzed with FlowJo v.9.4.11 (Tree Star).

### Flow cytometry analysis and FACS on explant cultures and embryonic tissues

Collagenase (0.0012 g/ml, Sigma-Aldrich) in PBS supplemented with 10% FCS (Omega Scientific) was employed to dissociate freshly isolated embryonic tissues or cultured explants. For flow cytometry cell sorting or AGM analyses, dissociated cells were stained with one or more of these antibodies: CD45.2-V500 (104) (562130, BD Biosciences), CD41-PerCP-eFluor710 (eBioMWReg30, 46-0411-82, eBioscience), CD43-FITC (eBioR2/60, 11-0431-82, eBioscience), VE-cadherin (CD144)-PE (11D4.1, 138009, BD Biosciences), CD45-FITC (30-F11, 11-0451-82, eBioscience), c-Kit-APC-eFluor780 (2B8, 47-1171-82, eBioscience). All antibodies were used at 1:200 dilution as previously described (Ganuza et al., 2018). DAPI staining was used to gate live events. Cell sorting was performed on a BD FACSAria III SORP (Special Order Research Product) and analysis was performed on an LSR Fortessa (both BD Biosciences). Data were analyzed as described above.

### Confocal microscopy and enumeration of clusters

E10.5 embryos were isolated and fixed in 4% paraformaldehyde (Electron Microscopy Sciences) overnight at 4°C. Embryos were processed, stained and tissue-cleared using the protocol previously described by the Dzierzak laboratory (Yokomizo et al., 2012). Embryos were stained with biotinylated rat anti-mouse CD31 antibody (MEC13.3, 553371, BD Biosciences), rabbit anti-c-Kit (clone D13A2, 3074, Cell Signaling Technology), goat anti-rabbit Alexa Fluor 647 secondary antibody (1:2500; clone A21245, A-21245, Thermo Fisher Scientific) and Alexa Fluor 488 goat anti-rat IgG (H+L) (A-11006, Invitrogen). Embryos were imaged on a Zeiss LSM780 confocal microscope using a 40×, 1.1 NA water immersion lens. Images were processed and stitched using ZEN 2012 software (Zeiss) and Imaris Stitcher and clusters were analyzed and counted with ZEN 2012 software.

### CLR analyses

CLR was applied as previously described (McKinney-Freeman et al., 2012) to unveil common putative transcriptional regulators of HSC specification. Gene modules were previously classified as specifying modules if their expression was significantly higher in hematopoietic stem cell precursors during HSC specification (McKinney-Freeman et al., 2012). Genes assigned to each of the specifying modules (ME-2, 17, 19, 26, 38, 40, 46, 56, 59) can be found in a previously published study (McKinney-Freeman et al., 2012). Genes highly connected upon CLR analysis were defined as ‘hub’ genes.

### Analysis of bulk mRNA and single-cell RNA-sequencing data from publicly available datasets

Expression levels of *Runx1*, *Nr4a1* and *Nr4a2* mRNA from populations of interests were extracted from the StemSite portal (McKinney-Freeman et al., 2012). Single-cell RNA-sequencing expression data for *Runx1*, *Nr4a1* and *Nr4a2* mRNA were extracted from figure S3 of Zhou et al. (2016).

### Real-time qRT-PCR

Total RNA was isolated from 600-25,000 VE-cadherin^−^CD45^−^, VE-cadherin^+^CD45^−^ and VE-cadherin^+^CD45^+^cells (QIAGEN RNeasy Micro Kit). RNA was amplified and reversed transcribed into cDNA employing RNA was amplified employing the NuGEN Ovation Pico WTA V2 system (NuGEN Technologies). Real-time qRT-PCR was performed using Fast SYBR Green Master Mix (Applied Biosystems) on an ABI StepOnePlus thermal cycler (Applied Biosystems) according to the manufacturer's instructions. PCR program: 95°C for 20″ (95°C for 1″ and 60°C for 20″) ×40, (melt curve) 95°C for 15″, 60°C for 15″, and 95°C for 15″. *Tbp* mRNA expression levels were used to compensate differences in cDNA input. The ΔΔCt method was applied to calculate changes in gene expression. Primers were used at 0.4 μM.

Primer sequences were: *Nr4a1*-Fw1 (5′-TTGAGTTCGGCAAGCCTACC3′), *Nr4a1*-Rv1 (5′-GTGTACCCGTCCATGAAGGTG-3′), *Nr4a2*-Fw2 (5′-ACACACACACCTTAATGGGACCCT-3′), *Nr4a2*-Rv2 (5′-CATGCCACCCACGCAACATTTAGT-3′), *Tbp*-F6 (5′-GAAGAACAATCCAGACTAGCAGCA-3′), *Tbp*-R6 (5′-CCTTATAGGGAACTTCACATCACAG-3′), *Runx1*-Fw2 (5′-GCAGGCAACGATGAAAACTACT-3′), *Runx1*-Rv2 (5′-GCAACTTGTGGCGGATTTGTA-3′).

### Statistics and reproducibility

For analyses, summary statistics, including mean and standard deviation are reported. Two-sample *t*-tests, exact Wilcoxon rank sum tests, Kruskal–Wallis tests or Mann–Whitney tests were used to test for differences between two groups depending on the normality of the data, which was assessed by the Shapiro–Wilk test. The false discovery rate (FDR) method described by Benjamini and Hochberg (1995) was used to correct for multiple comparisons at a level of 0.05. Otherwise, *P*<0.05 was considered statistically significant. Analyses were conducted in R v.3.3.1. Sample size and number of experiment replicates are detailed in each figure legend.

### CosMx SMI slide preparation

Samples in this study were processed following the guidelines outlined in the CosMx SMI Manual Slide Preparation for RNA Assays user manual (MAN-10184-02, NanoString). Embryos were fresh frozen (FF) in Tissue-Tek O.C.T. Compound with isopentane in liquid nitrogen. Isopentane was maintained at a temperature between −80°C and −100°C. FF samples were cut to 10 µm thickness and placed on a Superfrost Plus Premium Microscope Slide (VWR, 48311-703). In total, two slides containing nine *Nr4a1^+/+^Nr4a2^+/+^* sections each and two slides containing nine *Nr4a1^−/−^Nr4a2^−/−^* sections each were selected for CosMx. Slides were stored at −80°C until they were submerged in 10% neutral buffered formalin (NBF) (EMS Diasum, 15740) at 4°C for 2 hours. Slides were then washed three times in 1× PBS (Thermo Fisher Scientific, AM9625, AM9922) for 2 min each, then baked vertically at 60°C for 30 min. Once the tissue was fixed to the slide, FF samples were washed three times in 1× PBS for 5 min, once in 4% SDS (Thermo Fisher Scientific, AM9822) for 2 min, and three times in 1× PBS for 5 min each. Slides were then rehydrated with a 50% ethanol wash, a 70% ethanol wash, and two 100% ethanol washes for 5 min each. Next, samples were incubated in 1× Target Retrieval Solution (NanoString CosMx FF Slide Preparation Kit, RNA) at 100°C in a steamer for 8 min for heat-induced epitope retrieval. Immediately following this, slides were dipped in DEPC-treated water (Thermo Fisher Scientific, AM9922) for 15 s, washed in 100% ethanol for 3 min, and subsequently left to dry on the benchtop on a clean Kimwipe at room temperature for 30 min. After drying, the tissue was incubated at room temperature with 400 µl of digestion buffer, which contained a combination of Proteinase K and Protease A (NanoString CosMx FF Slide Preparation Kit, RNA), for 15 min followed by two 1× PBS washes for 5 min each. Next, slides were incubated at room temperature with fiducials (NanoString CosMx FF Slide Preparation Kit, RNA) at a working concentration of 0.00015% for 5 min protected from light, followed by one 5 min 1× PBS wash to remove excess fiducials. Slides then underwent a 1 min incubation in 10% NBF followed by two 5 min NBF STOP buffer washes, and one 5 min 1× PBS wash. A 100 mM NHS-acetate mixture was prepared using sulfo NHS-acetate powder (Fisher Scientific, 26777) and NHS-Acetate buffer (NanoString CosMx FF Slide Preparation Kit, RNA), which was then applied to the tissue and left to incubate protected from light at room temperature for 15 min. The slides were washed in 2× saline sodium citrate (SSC) (Thermo Fisher Scientific, AM9763, AM9922) twice for 5 min each after NHS-acetate incubation.

The CosMx Mouse Universal Cell Characterization panel contains 950 mouse gene targets with an additional set of 50 custom targets, chosen specifically for this study. The NanoString and custom *in situ* hybridization (ISH) probes were denatured at 95°C for 2 min and immediately transferred to ice for 1 min. The ISH probes were then combined with RNase inhibitor, Buffer R, and DEPC-treated water and applied to the slides as they were placed one-by-one into a hybridization chamber. Coverslips were applied to the slides to prevent loss of the probe mix during overnight hybridization. The slides were incubated overnight for 17 h protected from light at 37°C. After overnight probe hybridization, slides were washed in 2× SSC to remove coverslips and subsequently washed twice in a 50% formamide (Thermo Fisher Scientific, AM9342) solution with 2× SSC for 25 min at 37°C to remove off-target probes. Formamide washes were followed by two 2× SSC washes for 2 min each at room temperature. The DAPI stock solution (NanoString, CosMx Mouse Universal Cell Segmentation Kit) was diluted 1:40 and applied to each slide for 15 min at room temperature. Following nuclear staining, slides were washed in 1× PBS for 5 min before a cocktail of antibodies including CD298/B2 M, PanCK, and CD45 (NanoString, CosMx Mouse Universal Cell Segmentation Kit) was applied to tissue for 1 h at room temperature. Slides were then washed in 1× PBS for 5 min for a total of three washes to remove off-target antibodies and stored overnight at 4°C in 2× SSC. In final preparations to load the slides onto the CosMx instrument, a flow cell was applied to each slide. Slides were then loaded onto the CosMx SMI per manual MAN-10161-05, the CosMx SMI Instrument User Manual, using pre-bleaching profile C. For the cell segmentation, we used Configuration=C and Probability Threshold=−6. Preview scans of each slide were taken by the CosMx SMI, and field of views were selected to encompass the entirety of each embryo. Some sections peeled off during CosMx processing, and some were excluded from the analysis for being damaged in the areas of interest.

### CosMx data processing and analysis

Primary data were processed using NanoString's proprietary AtoMx software (v.1.3.2), which resulted in three main files, an expression matrix in the format of cell-by-gene, a .csv file containing information for each transcript identified, and a metadata file with information about each cell.

Seurat (v.5.0.3) (Hao et al., 2024) was used for downstream analysis. Cells with fewer than ten transcripts, fewer than ten unique genes and an area of more than 500 µm^2^ were excluded for downstream analysis. Principal component analysis using 50 principal components were calculated using all genes available in the CosMx panel (*n*=1000), the optimal number of principal components selected for building the neighborhood graph was 15. For unsupervised clustering, Insitutype (Danaher et al., 2022 preprint) was used, specifically the function ‘insitutype’; we used the negative control probe values of CosMx for the linear modeling and the following parameters: *n*_phase1=200, n_phase2=500, n_phase3=2000, n_starts=1, max_iters=5). We performed selected specific regions for each embryo section based on areas of interest and ran the unsupervised clustering on all *Nr4a1*^−/−^*Nr4a2*^−/−^ sections together, and all *Nr4a1*^+/+^*Nr4a2*^+/+^ sections together, which resulted in five main clusters in each group. A one-versus-all differential expression approach was employed to obtain gene markers per cluster, which was used in addition with canonical markers for cell annotation.

We used UCell (Andreatta and Carmona, 2021) to calculate the score for Notch signaling pathway, using 37 genes from the Gene Ontology term GO:0007219 (Notch signaling pathway), which overlaps with the CosMx panel. All statistical tests were performed using R (v.4.3.1).

## Supplementary Material



10.1242/develop.201957_sup1 Supplementary information

Table S1. Source data file. Each tab in the spreadsheet contains source data for the indicated figures including Fig. 1B, Fig. 1C, Fig. 1D, Figs. 2B-C, Figs. 2D-E, Figs. 3B-C, Figs. 4B-C, Fig. 5C, Fig. 5D-E and Suppl. Fig. 2. Please see figure legends within each Figure.

Table S2. List of genes used for CosMx analysis. Contains the 950 core genes included in the CosMx Mouse Universal Cell Characterization RNA Panel and 50 custom genes.
