# Supplementary material for: NR4A1 and NR4A2 orphan nuclear receptors regulate endothelial-to-hematopoietic transition in mouse hematopoietic stem cell specification
Source: Development. 2024 Nov 26;151(22):dev201957. doi: 10.1242/dev.201957 (PMC11634030; doi:10.1242/dev.201957)
Supplement: Supplementary information [file develop-151-201957-s1.pdf]

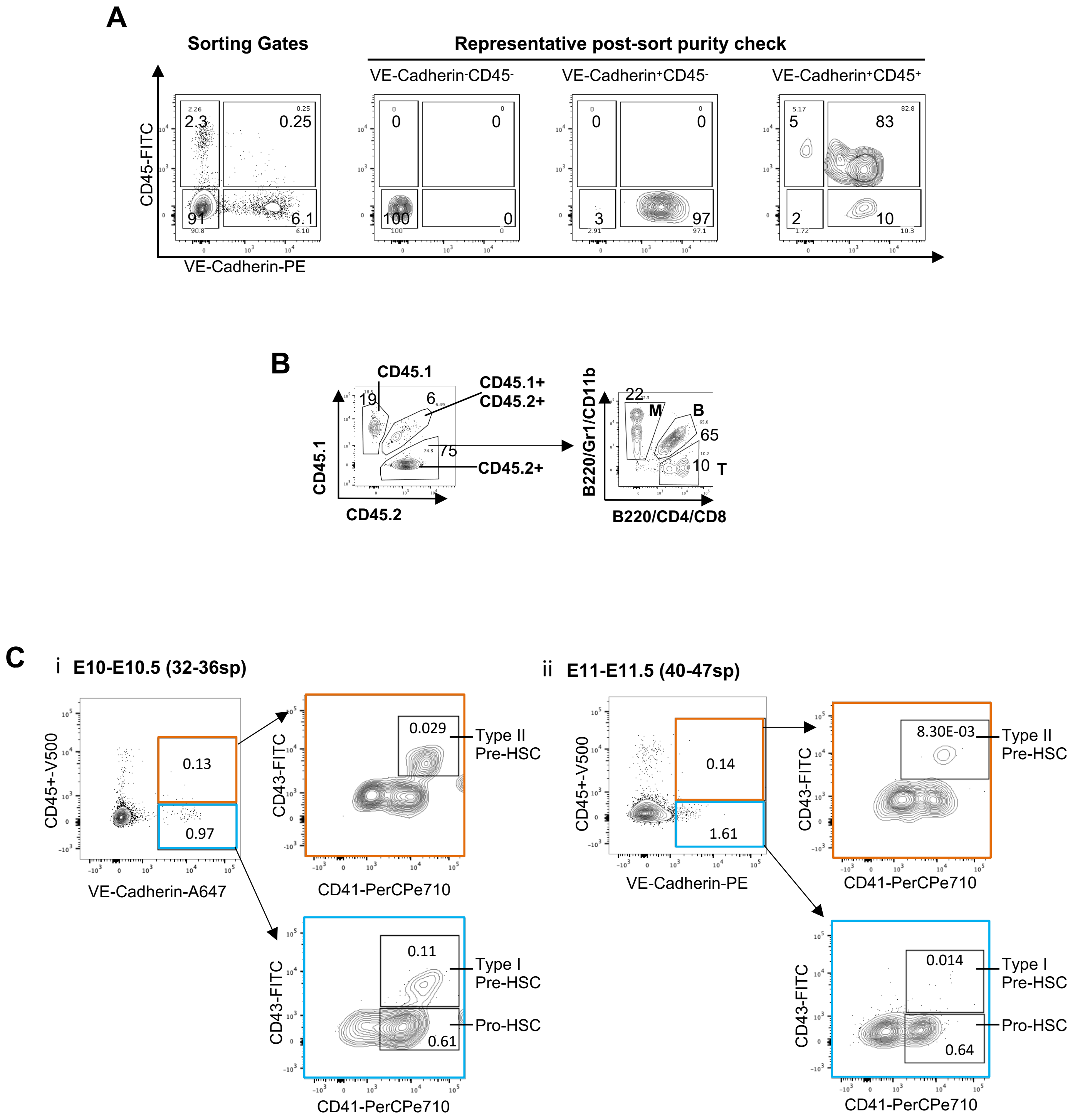

Fig. S1. Gating strategies. **A)** Representative gating strategy for VE- Cadherin$^-$CD45$^-$, VE-Cadherin$^+$CD45$^-$ and VE-Cadherin$^+$CD45$^+$. Post-sort purity checks are shown. Related to Fig. 1D. **B)** Representative flow cytometry analysis of the PB of a transplant recipient 16 weeks post-transplant. Related to Figs 2B, 2D, 4B. (** $p < 0.01$, * $p < 0.05$). **C)** Flow cytometry analysis gating strategies of E10.5 (i) and E11-E11.5 (ii) pro- HSCs (VE$^+$CD41$^{+/lo}$CD43$^-$CD45$^-$), type I (VE$^+$CD41$^{+/lo}$CD43$^+$CD45$^-$) and type II pre- HSCs (VE$^+$CD41$^{+/lo}$CD43$^+$CD45$^+$).

Development • Supplementary information

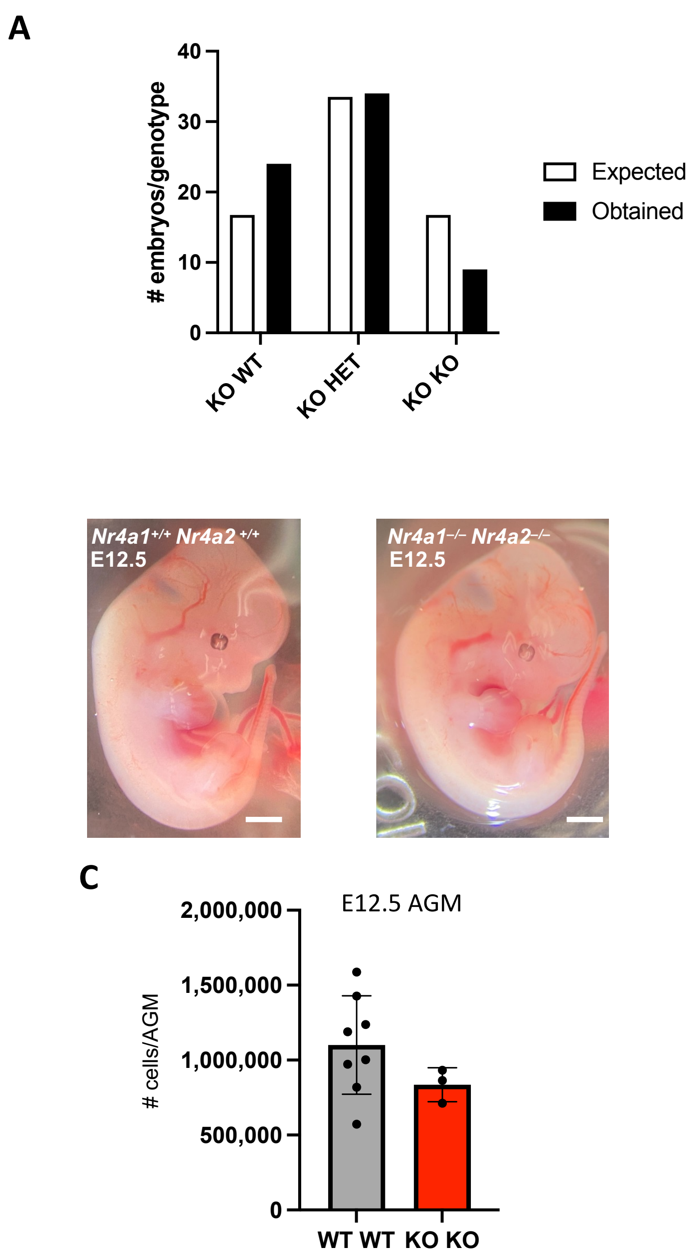

Fig. S2. Nr4a1-/- Nr4a2-/- embryos are morphologically normal at E12.5.

A) Number of embryos expected vs obtained per genotype. Chi-square test for goodness of fit found significant differences p=0.0345. B) Representative images *Nr4a1*$^{+/+}$*Nr4a2*$^{+/+}$ and *Nr4a1*$^{-/-}$*Nr4a2*$^{-/-}$ E12.5 embryos. C) AGM cellularity of E12.5 *Nr4a1*$^{+/+}$*Nr4a2*$^{+/+}$ (n=2 independent experiments) vs *Nr4a1*$^{-/-}$*Nr4a2*$^{-/-}$ (n=2). Embryos were acquired over 4 independent experiments). Scale bar: 1mm.

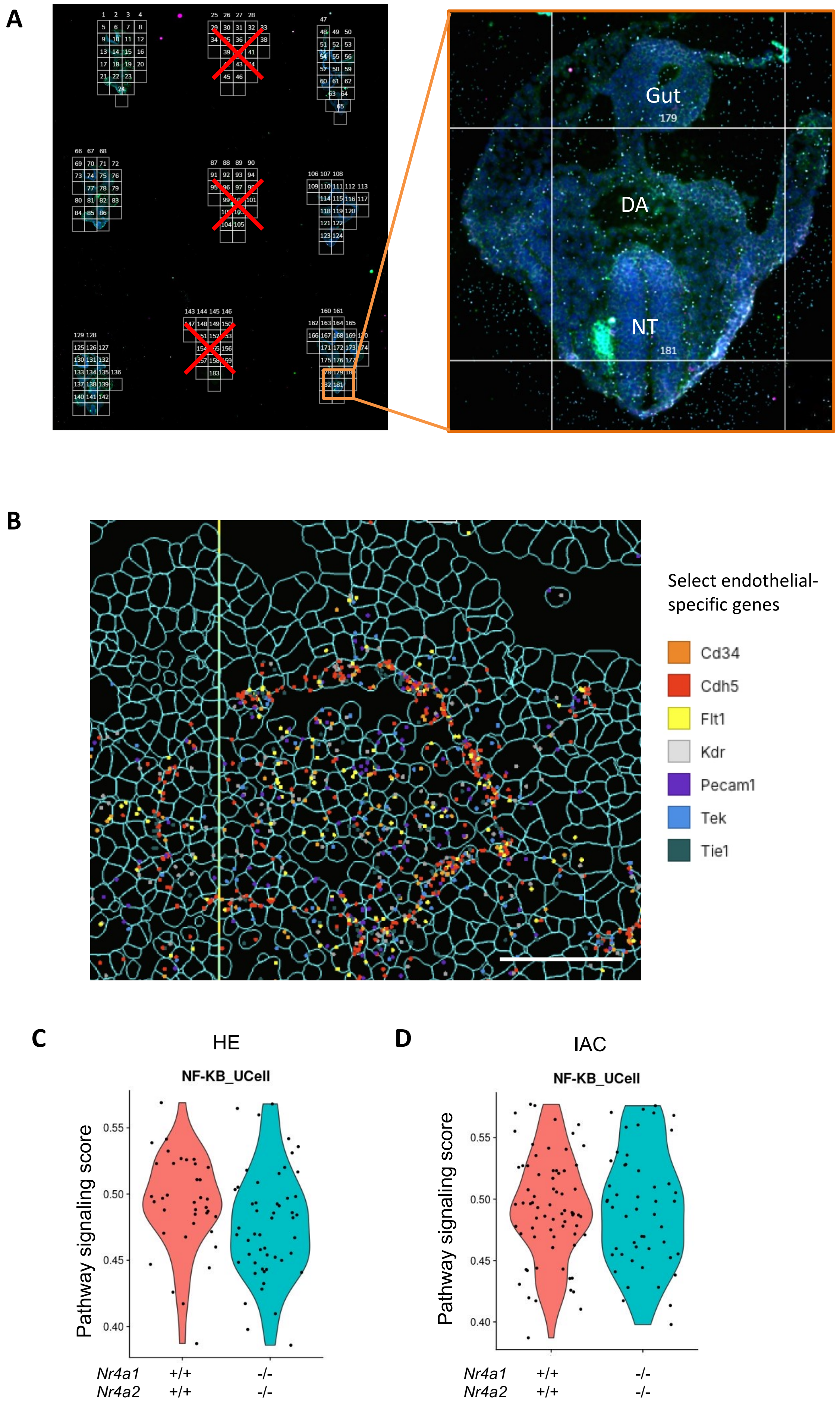

Development • Supplementary information

Fig. S3. CosMx SMI clustering analysis.

A) Representative example of a slide that was used for CosMx SMI. 9 sections were taken per slide (left panel). Red crosses mark sections that either washed off or were damaged during processing. A close-up view of the orange square is shown on the right panel and includes the AGM region and surrounding tissue; dorsal aorta (DA), neural tube (NT) and gut are shown. Sections were stained with DAPI (blue) and a cocktail of antibodies containing CD298/B2M, PanCK, and CD45 (green). **B)** Representative example of a section showing a selection of endothelial transcripts as well as cell segmentation. The vertical green line separates 2 adjacent fields of view (FOVs). Scale bar: 100µm. **C-D)** Violin plots showing NFκB pathway signaling score in **C)** HE (p=0.06868) and **E)** IACs (p=0.9606) between *Nr4a1*$^{+/+}$*Nr4a2*$^{+/+}$ and *Nr4a1*$^{-/-}$ *Nr4a2*$^{-/-}$ embryos. A two-tailed unpaired t-test was used.

Table S1. Source data file. Each tab in the spreadsheet contains source data for the indicated figures including Fig. 1B, Fig. 1C, Fig. 1D, Figs. 2B-C, Figs. 2D-E, Figs. 3B-C, Figs. 4B-C, Fig. 5C, Fig. 5D-E and Suppl. Fig. 2. Please see figure legends within each Figure.

Available for download at
https://journals.biologists.com/dev/article-lookup/doi/10.1242/dev.201957#supplementary-data

Table S2. List of genes used for CosMx analysis. Contains the 950 core genes included in the CosMx Mouse Universal Cell Characterization RNA Panel and 50 custom genes.

Available for download at
https://journals.biologists.com/dev/article-lookup/doi/10.1242/dev.201957#supplementary-data

Development • Supplementary information
