# Supplementary material for: NR4A1 and NR4A2 orphan nuclear receptors regulate endothelial-to-hematopoietic transition in mouse hematopoietic stem cell specification
Source: Development. 2024 Nov 26;151(22):dev201957. doi: 10.1242/dev.201957 (PMC11634030; doi:10.1242/dev.201957)
Supplement: Supplementary file 1 [file develop-151-201957_review_history.pdf]

NR4A1 and NR4A2 orphan nuclear receptors regulate endothelial-to-hematopoietic transition in mouse hematopoietic stem cell specification

Diana Sá da Bandeira, Chris D. Nevitt, Felipe Segato Dezem, Maycon Marção, Yutian Liu, Zakiya Kelley, Hannah DuBose, Ashley Chabot, Trent Hall, Claire Caprio, Victoria Okhomina, Guolian Kang, Jasmine Plummer, Shannon McKinney-Freeman, Wilson K Clements and Miguel Ganuza
DOI: 10.1242/dev.201957

Editor: Hanna Mikkola

Review timeline
Original submission: 10 May 2023
Editorial decision: 5 June 2023
First revision received: 28 September 2024
Accepted: 14 October 2024

Original submission

First decision letter

MS ID#: DEVELOP/2023/201957

MS TITLE: NR4A1 and NR4A2 orphan nuclear receptors regulate endothelial to hematopoietic transition in mouse hematopoietic stem cell specification

AUTHORS: Diana Sa da Bandeira, Chris D Nevitt, Victoria Okhomina, Ashley Chabot, Trent Hall, Claire Caprio, Guolian Kang, Wilson K Clements, Shannon McKinney-Freeman, and Miguel Ganuza

I have now received all the referees' reports on the above manuscript, and have reached a decision. The referees' comments are appended below, or you can access them online: please go to BenchPress and click on the 'Manuscripts with Decisions' queue in the Author Area.

As you will see, the referees express considerable interest in your work, but have some significant criticisms and recommend a substantial revision of your manuscript before we can consider publication. If you are able to revise the manuscript along the lines suggested, which may involve further experiments, I will be happy receive a revised version of the manuscript. Your revised paper will be re-reviewed by one or more of the original referees, and acceptance of your manuscript will depend on your addressing satisfactorily the reviewers' major concerns. Please also note that Development will normally permit only one round of major revision. If it would be helpful, you are welcome to contact us to discuss your revision in greater detail. Please send us a point-by-point response indicating your plans for addressing the referee’s comments, and we will look over this and provide further guidance.

Please attend to all of the reviewers' comments and ensure that you clearly highlight all changes made in the revised manuscript. Please avoid using 'Tracked changes' in Word files as these are lost in PDF conversion. I should be grateful if you would also provide a point-by-point response detailing how you have dealt with the points raised by the reviewers in the 'Response to Reviewers' box. If you do not agree with any of their criticisms or suggestions please explain clearly why this is so.

© 2024. Published by The Company of Biologists under the terms of the Creative Commons Attribution License (https://creativecommons.org/licenses/by/4.0/).

<u>Reviewer 1</u>

Advance summary and potential significance to field

In this manuscript, Sa da Bandiera et al have investigated the effect of losing Nr4a1 and Nr4a2 expression during haematopoietic stem cell formation predominantly in the AGM region. To my knowledge, this has not been performed previously. The data in this manuscript are clearly presented, the experiments appear to be rigorously performed, and the conclusions are well supported by the data. The authors have provided interesting and convincing data that demonstrates that the early stages of HSC formation require normal Nr4a1 and Nr4a2 expression. As stated by the authors, this insight provides valuable new knowledge about how haematopoietic stem cells are produced.

Comments for the author

1. Could the authors provide relevant background about Nr4a1-/- and Nr4a2-/- mice, particularly the double knockout mice? For example, are Nr4a1-/-;Nr4a2-/- mice viable? Do Nr4a1-/-;Nr4a2-/- mice development normally (especially up to E11.5)?.

This information is essential to follow the study general, and also to interpret the meaning of the of values presented in Figure 4 (e.g., is the total cellularity of the AGM regions the same between the genotypes?). Representative examples of the embryos, AGM region, and analysis of cell numbers would be useful.

2. (a) Given that the decrease in IACs appeared to be restricted to the aorta of Nr4a1-/-;Nr4a2-/- mice (Figure 3B), would it be more accurate to conclude that these receptors have a role in HSC development in the AGM region?

(b) Can the authors provide an explanation for why IAC formation would be specifically reduced in the aorta versus the UC/VC? Does the pattern of Nr4a1 and Nr4a2 expression provide any insight?

(c) Are there HSC defects in other Nr4a1-/-;Nr4a2-/- E11.5 tissues (e.g. the yolk sac, peripheral blood, placenta, UC or VC) or is the phenotype restricted to the AGM region?

3. To understand whether the Nr4a axis is AGM region specific or a mechanism regulating general HSC production, could the authors provide information about the size of the HSC pool in the later fetal liver (e.g. at E14.5)?

4. Regarding data in Figure S2. The secondary reconstitution data are very interesting, especially with cells on a Nr4a1-/-;Nr4a2+/- background. What does this tell us about the role of these genes in the HSC lineage: are they required for HSC function in the adult, or is this a consequence of poorly produced HSCs?
Insight might be provided from secondary reconstitution studies using E10.5 explant cultures?

The reviewer acknowledges that speculating about this point (rather than performing additional experiments) would be adequate.

5. Given the arguments made on Pg17. Have the authors investigated if diminished NOTCH signalling/diminished expression of NOTCH1 target genes occurs in the relevant cell types of Nr4a1-/-;Nr4a2-/- mice?

6. Pg9 line 159 – 161: “These data show that Nr4a genes are specifically upregulated during EHT…”. This implies a specific expression of these genes during EHT. This is problematic because the investigation is from a narrow set of cells. It could be said that rather being specifically upregulated during EHT, these genes are expression in haematopoietic/HSC lineages more than the endothelium. This also applies to Pg9 line 169.
Perhaps this could be rephrased.

7. There appears to be a stitching issue with the Nr4a1-/-;Nr4a2-/- image in Figure 3A.

© 2024. Published by The Company of Biologists under the terms of the Creative Commons Attribution License (https://creativecommons.org/licenses/by/4.0/).

8. Pg16 lines 313 – 315: "Interestingly, enforced expression of Nr4a2 in mouse hematopoietic progenitor adult cells drives early hematopoietic progenitors into quiescence through upregulation of the cell-cycle inhibitor INK4C (p18) (Sirin, Lukov, Mao, Conneely, & Goodell, 2010). When transplanted into lethally irradiated mice, these cells can home to bone marrow but do not regenerate the blood (Sirin et al., 2010). Accordingly, we observed an accumulation of HSC in adult Nr41-/-Nr4a2+/- mice, revealing a putative block in differentiation (data not shown)."

I struggle to following the logic of this point: if overexpression of Nr4a2 results in a block at the HSPC level why would one expect to observe a block in differentiation in Nr41-/-Nr4a2+/-?

Apologies if this an issue with the reviewer rather than the text.

9. Introduction of references to "data not shown" on Pg 16 and Pg19: if the authors wish to refer to new data it really should be included for scrutiny (perhaps as supplemental figures) instead of being referred to as "data not shown". Typically, this should be introduced in the main text rather than in the discussion.

Reviewer 2

Advance summary and potential significance to field

The manuscript by Sa da Banderia et al. focuses on HSC emergence during endothelial to hematopoietic transition (EHT), aiming to characterize new regulators of this process, as previously described with Runx1, considered a master regulator of EHT. The authors identify the Nr4a family as putative regulators of multiple stages in HSC specification, and then test the function of these transcriptional regulators during EHT.
The topic of study is very relevant, as it is critical to understand developmental processes related to hematopoietic emergence and its implications in adult homeostasis and disease. However, the authors need to address substantial issues in this manuscript to guaranty the robustness of their findings.

Comments for the author

The biggest limitation of the study as presented is the high variability in some of the results, which makes their interpretation very difficult.
From data on Figure 2 it seems clear that Nr4a1/2 double KO have the strongest phenotype, not being able to mature into engrafting HSCs after explant culture.
This, and the imaging data on Figure 3 are the most robust and easy-to-interpret results in the entire paper. But then Figure 4 stains Pre and ProHSCs and there is substantial variability. I fear that variability in dissection and cell isolation has created a lot of error that needs to be corrected. The best option is obviously to calculate and plot absolute numbers of cells per embryo. But if this is hard, an alternative is to use a relative measure based on a positive gate e.g. % of all Vcad+ (this makes the reasonable assumption that the number of Vcad+ stayed equal across embryos). Another challenging thing to interpret in Figure 4 is the "gene dosage" effect. The authors include many different mouse strains lacking either 1 or both copies of Nr4a1 and/or Nr4a2, but then some combinations are missing and other combinations have very few and wildly different numbers of replicates. This quickly becomes impossible to interpret (and hard to perform statistics for). I would advice to increase the number of replicates across genotypes, or just stick to the ones that have enough replicates to achieve robust conclusions (WT, and the series of Nr4a1-/-, which are the last 3 columns).

The second biggest concern is the confusing results on Figure 5, which strikingly contrast with Figure 2. I have issue with trying to compare them side-by-side with E11.5 transplant data, as the authors attempt (in the results and discussion).

First, the success of the cultures is significantly better after explant cultures in Figure 5, with most mice being able to engraft even if fewer starting material is transplanted (0.3 EE/mouse). It is my

© 2024. Published by The Company of Biologists under the terms of the Creative Commons Attribution License (https://creativecommons.org/licenses/by/4.0/).

understanding that E11 AGMs can also be processed in the same manner. It would be important to know whether the cultures also have the capacity to induce hemogenesis from Nr4a1/2 null at E11.

An alternative interpretation of the data is that Nr4a null HSCs at E11.5 have increased Nfkb signaling, matured faster and have already migrated out of the AGM (something that could also fit with the E10 data). One way to test this would be to test fetal liver FACS and transplants at E11.5.

In general, these seems to be a lack of attempt at describing the mechanisms and genes being regulated by Nr4a1/2. Considering these are transcriptional regulators, the most straightforward experiment would be to perform some sort of ATACseq or RNAseq experiment at E10-11. Considering the heterogeneity in clusters at E10, perhaps the best option would be to perform single-cell profiling comparing WT and Nr4a1/2-null. The most pressing question, considering the results in Figure 2, is if Nr4a1/2-null AGM lacks expression of Runx1 (placing it upstream of the EHT pathway). These could be compared with Freire et al. 2018 to see if NfkB signaling (a regulator of EHT) is also altered.

Minor concerns:

Figure S2D is not mentioned in the figure.

Supplementary 2A-C figures could be placed together with the main figure 2 as secondary transplant aim to demonstrate the proper functionality of donor cells.
WT cells are not shown as control.

Line 215 lacks Fig. 3A call and line 217 lacks Fig. 3B/C call.

Magnification value size in Fig. 3A should be added in the main figure to understand the difference between column A and B.
Line 225 should specify 4A.
Unclear why authors do not study Nr4a3 (together with 4a1 and 4a2). Is it expressed? No? This should be shown in Figure 1, perhaps.

First revision

<u>Author response to reviewers' comments</u>

Sa da Bandeira *et al*. Response to Reviewers

We would like to thank the Reviewers for their useful and constructive comments, and for the opportunity to address their questions. Please find below point-by-point response to the comments raised and how we have addressed them.

Reviewer 1

Advance Summary and Potential Significance to Field: In this manuscript, Sa da Bandeira et al have investigated the effect of losing Nr4a1 and Nr4a2 expression during haematopoietic stem cell formation, predominantly in the AGM region. To my knowledge, this has not been performed previously. The data in this manuscript are clearly presented, the experiments appear to be rigorously performed, and the conclusions are well supported by the data. The authors have provided interesting and convincing data that demonstrates that the early stages of HSC formation require normal Nr4a1 and Nr4a2 expression. As stated by the authors, this insight provides valuable new knowledge about how haematopoietic stem cells are produced.

Reviewer 1 - Comments for the Author:
1) Could the authors provide relevant background about Nr4a1-/- and Nr4a2-/- mice, particularly the double knockout mice? For example, are Nr4a1-/-;Nr4a2-/- mice viable? Do Nr4a1-/-;Nr4a2-/- mice development normally (especially up to E11.5)?.
This information is essential to follow the study general, and also to interpret the meaning of the of values presented in Figure 4 (e.g., is the total cellularity of the AGM regions the same between

© 2024. Published by The Company of Biologists under the terms of the Creative Commons Attribution License (https://creativecommons.org/licenses/by/4.0/).

the genotypes?). Representative examples of the embryos, AGM region, and analysis of cell numbers would be useful.

We agree with the Reviewer and have added the following information to the main text (results section, lines 179-183):
"Although *Nr4a1*$^{-/-}$*Nr4a2*$^{-/-}$ embryos were not observed at Mendelian ratios (Supplementary Fig. 2A), all the *Nr4a1*$^{-/-}$*Nr4a2*$^{-/-}$ embryos we obtained had a visible heartbeat and their morphology (Supplementary Fig. 2B) and AGM cellularity were normal at E12.5 (Supplementary Fig. 2C), suggesting that these embryos have no overt defects at least until after E12.5".
We have included the Mendelian ratios graph, representative images of the embryos and AGM cellularity at E12.5 in Supplementary Fig. 2 (see below). Importantly, we did not observe any dead or resorbing *Nr4a1*$^{-/-}$*Nr4a2*$^{-/-}$ embryos in any embryonic litter, suggesting that any effects of *Nr4a1* and *Nr4a2* deletions on embryo viability happens a lot earlier, precluding the detection of resorbed embryos.

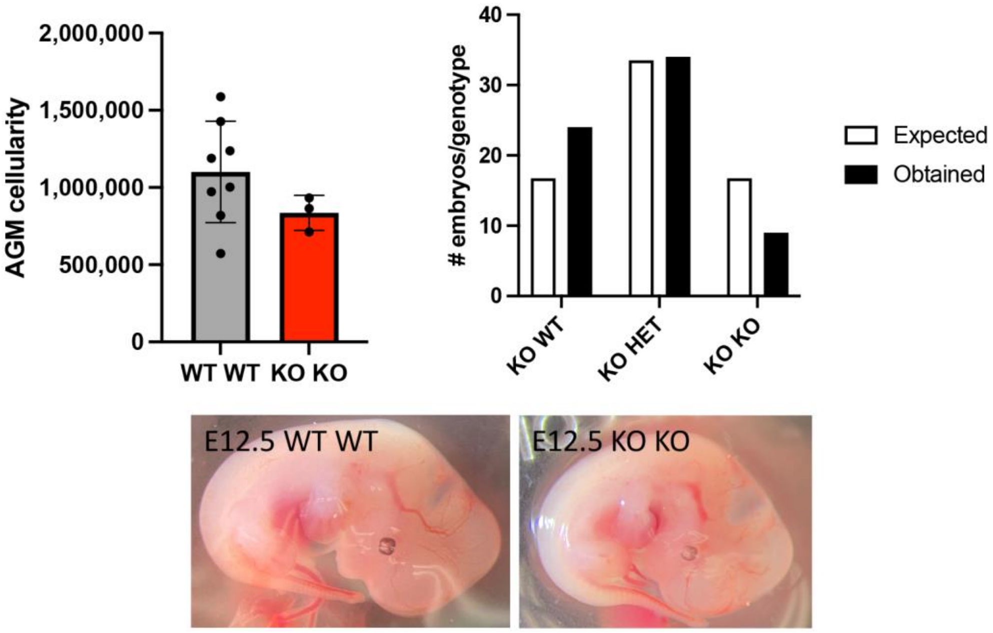

2.(a) Given that the decrease in IACs appeared to be restricted to the aorta of *Nr4a1*$^{-/-}$; *Nr4a2*$^{-/-}$ mice (Figure 3B), would it be more accurate to conclude that these receptors have a role in HSC development in the AGM region?

We agree with the Reviewer and have rephrased our conclusions. We now wrote: "Interestingly, we observed a significant decrease in the numbers of more mature pre-HSCs-type II in E11.5 *Nr4a1*$^{-/-}$*Nr4a2*$^{-/-}$ embryos, suggesting an impaired HSC specification process in the AGM (Fig. 3D-E)." (Lines 237-239)

(b) Can the authors provide an explanation for why IAC formation would be specifically reduced in the aorta versus the UC/VC? Does the pattern of Nr4a1 and Nr4a2 expression provide any insight?

We thank the Reviewer for their comment. Although understanding the differences in EHT in the aorta vs. arteries is interesting, we consider this beyond the scope of this study. We see a progressive reduction in the number and size of the clusters in the umbilical artery together with a reduction in Nr4a gene dosage. We are unsure why this reduction is not observed in the vitelline artery. In both umbilical and vitelline arteries, we found a larger variability than in the aorta. It would be interesting to see NR4A1 and 2 expression in IAC in the aorta vs. arteries but we have unfortunately had technical difficulties with these antibodies. Since we haven't carried out any specific functional assays with the umbilical and vitelline arteries in this study, we have removed these data and focused solely on the AGM region.

© 2024. Published by The Company of Biologists under the terms of the Creative Commons Attribution License (https://creativecommons.org/licenses/by/4.0/).

(c) Are there HSC defects in other Nr4a1-/-;Nr4a2-/- E11.5 tissues (e.g. the yolk sac, peripheral blood, placenta, UC or VC) or is the phenotype restricted to the AGM region?

We thank the Reviewer for their comment. Since blood circulation starts at E8.5, HSC functional defects detected in the AGM at E11.5 will likely be observed in other organs. Due to a limitation in the number of mice, we have focused on analyzing the HSC defects in the E12.5 FL, as it becomes the major hematopoietic organ from E12.5 onward. We found that the frequency of HSCs within the LSK population was unchanged with the loss of *Nr4a1* and *Nr4a2*. However, because the frequency of LSK is lower when gated from the parent population (Lin-), we could conclude that the absolute number of HSCs ($CD48^{-}CD150^{+}$ LSK) must be lower.
On the other hand, if we plot all the data from single live cells, we do not find differences in LSKs nor HSCs. Because these data are inconclusive, we have decided not to add them to the manuscript. But if the Reviewer thinks we should, we will add it as a supplementary figure and mention the unclear results.
We can conclude, however, that the AGM defects we observe in *Nr4a1*$^{-/-}$;*Nr4a2*$^{-/-}$ embryos is not due to a premature migration of HSPCs to the fetal liver.
% of LSK and HSCs from parent populations:

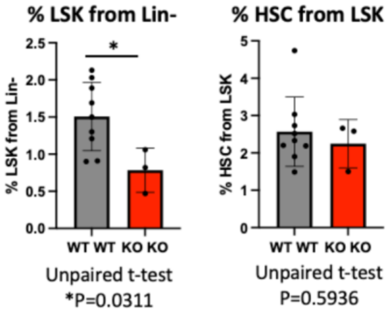

% LSK and HSCs from single live cells:

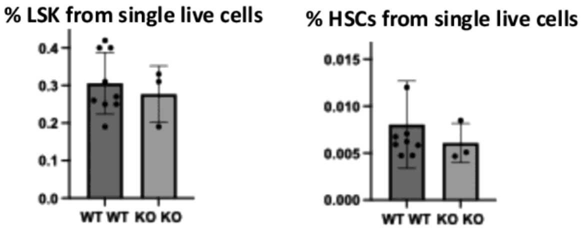

3. To understand whether the Nr4a axis is AGM region specific or a mechanism regulating general HSC production, could the authors provide information about the size of the HSC pool in the later fetal liver (e.g. at E14.5)?

We thank the Reviewer for this insightful comment. Due to the difficulty in obtaining litters and embryos with the right genotypes, we interrogated the phenotypic HSC pool in the E12.5 FL instead of the E14.5 FL (see above). Our data suggest an AGM-specific defect, but we are unable to rule out with certainty that other hematopoietic cells and progenitors in other organs are not affected. It would be interesting in a subsequent study to determine whether these HSC specification defects can be overcome by maturation and/or expansion in the E14 FL.

4. Regarding data in Figure S2. The secondary reconstitution data are very interesting, especially with cells on a Nr4a1-/-;Nr4a2+/- background. What does this tell us about the role of these genes in the HSC lineage: are they required for HSC function in the adult, or is this a consequence of poorly produced HSCs? Insight might be provided from secondary reconstitution studies using E10.5 explant cultures?

© 2024. Published by The Company of Biologists under the terms of the Creative Commons Attribution License (https://creativecommons.org/licenses/by/4.0/).

The reviewer acknowledges that speculating about this point (rather than performing additional experiments) would be adequate.

We thank the Reviewer for their comment. In the previous Figure 4, where we plotted pro- and type I and II pre- HSCs across genotypes, we also found an arrest in pro-HSC maturation (significant accumulation of pro-HSCs) and a significant decrease in type II pre-HSCs in *Nr4a1*$^{-/-}$ *Nr4a2*$^{+/-}$ embryos. Based on this, we hypothesize that a single copy of Nr4a2 is sufficient to slightly compensate for the loss of *Nr4a1* and *Nr4a2* and obtain enough mature HSCs for primary reconstitution. But based on the secondary reconstitution data, these HSCs are not fully functional and tend to lose their potency over time. It would be very interesting in a subsequent study to study later HSC maturation and the relevance of Nr4a genes in adult hematopoiesis.

We have added the following paragraph to the discussion (lines 209-214):
"This defect in self-renewal may indicate that the emerged *Nr4a1*$^{-/-}$*Nr4a2*$^{+/-}$ HSCs are not fully functional and the exposure to a wild type adult bone marrow microenvironment is not able to rewire/rescue their developmental defect. Alternatively, *Nr4a1* and *Nr4a2* genes may be required in adult hematopoiesis to maintain HSC function. Further investigations employing conditional knock-out Nr4a alleles would be required to formally address this."

5. Given the arguments made on Pg17. Have the authors investigated if diminished NOTCH signalling/ diminished expression of NOTCH1 target genes occurs in the relevant cell types of Nr4a1-/-;Nr4a2-/- mice?

We thank the reviewer for their suggestion. We have performed spatial transcriptomic analysis in E10 *Nr4a1*$^{-/-}$;*Nr4a2*$^{-/-}$ and *Nr4a1*$^{+/+}$;*Nr4a2*$^{+/+}$ embryos and found an increase in Notch signaling in *Nr4a1*$^{-/-}$;*Nr4a2*$^{-/-}$ intra-aortic cluster cells but not in hemogenic endothelial cells HECs, suggesting that although EHT initiates normally, it is unable to complete, due to a failure of repression of Notch signaling.
We have added these data in a new Fig. 5 and supplementary Fig. 3.
These new data is included and discussed in lines 272-325, 387-397 and 416-417.

6. Pg9 line 159 – 161: "These data show that Nr4a genes are specifically upregulated during EHT…". This implies a specific expression of these genes during EHT. This is problematic because the investigation is from a narrow set of cells. It could be said that rather being specifically upregulated during EHT, these genes are expression in haematopoietic/HSC lineages more than the endothelium. This also applies to Pg9 line 169. Perhaps this could be rephrased.

We agree with the Reviewer. We have rephrased this to:
-Lines 162-164: "These data show that *Nr4a* genes are upregulated in definitive hematopoietic cells, particularly in pre-HSCs type I and type II".
-Lines 172-173: "confirming that these genes are specifically upregulated in definitive hematopoietic cells.

7. There appears to be a stitching issue with the Nr4a1-/-;Nr4a2-/- image in Figure 3A.

We thank the Reviewer for this observation. We have re-stitched the image and replaced it in the figure.

8. Pg16 lines 313 – 315: "Interestingly, enforced expression of Nr4a2 in mouse hematopoietic progenitor adult cells drives early hematopoietic progenitors into quiescence through upregulation of the cell-cycle inhibitor INK4C (p18) (Sirin, Lukov, Mao, Conneely, & Goodell, 2010). When transplanted into lethally irradiated mice, these cells can home to bone marrow but do not regenerate the blood (Sirin et al., 2010). Accordingly, we observed an accumulation of HSC in adult Nr41-/-Nr4a2+/- mice, revealing a putative block in differentiation (data not shown)." I struggle to following the logic of this point: if overexpression of Nr4a2 results in a block at the HSPC level why would one expect to observe a block in differentiation in Nr41-/-Nr4a2+/-? Apologies if this an issue with the reviewer rather than the text.

We agree that this is a confusing observation and have removed it from the text.

© 2024. Published by The Company of Biologists under the terms of the Creative Commons Attribution License (https://creativecommons.org/licenses/by/4.0/).

9. Introduction of references to "data not shown" on Pg 16 and Pg19: if the authors wish to refer to new data it really should be included for scrutiny (perhaps as supplemental figures) instead of being referred to as "data not shown". Typically, this should be introduced in the main text rather than in the discussion.

We thank the Reviewer and have removed it from the text so that we only focus on HSCs during development.

Reviewer 2
Advance Summary and Potential Significance to Field: The manuscript by Sa da Banderia et al. focuses on HSC emergence during endothelial to hematopoietic transition (EHT), aiming to characterize new regulators of this process, as previously described with Runx1, considered a master regulator of EHT. The authors identify the Nr4a family as putative regulators of multiple stages in HSC specification, and then test the function of these transcriptional regulators during EHT. The topic of study is very relevant, as it is critical to understand developmental processes related to hematopoietic emergence and its implications in adult homeostasis and disease. However, the authors need to address substantial issues in this manuscript to guaranty the robustness of their findings.

Reviewer 2 Comments for the Author:
The biggest limitation of the study as presented is the high variability in some of the results, which makes their interpretation very difficult. From data on Figure 2 it seems clear that Nr4a1/2 double KO have the strongest phenotype, not being able to mature into engrafting HSCs after explant culture. This, and the imaging data on Figure 3 are the most robust and easy-to-interpret results in the entire paper. But then Figure 4 stains Pre and ProHSCs and there is substantial variability. I fear that variability in dissection and cell isolation has created a lot of error that needs to be corrected. The best option is obviously to calculate and plot absolute numbers of cells per embryo. But if this is hard, an alternative is to use a relative measure based on a positive gate, e.g. % of all Vcad+ (this makes the reasonable assumption that the number of Vcad+ stayed equal across embryos).

We thank the Reviewer for their observations. Respectfully, if the Reviewer focuses on the WT AGM controls in previous Figure 4B (new Figure 3D-E), one can appreciate that the variability between embryos is quite small. Thus, we interpret the high variability observed amongst *Nr4a*-deficient embryos as a phenotype related to reduced *Nr4a* gene dosage. We have recalculated the frequencies based on parent populations (as suggested) and indeed variability is still present (please see below), which we again interpret as a phenotype of these genetic models. In addition, the statistical analyses applied to these data accounts for this variability, and we consistently find a statistically significant arrest in pro-HSC development and a reduction in type II pre-HSCs when looking at the percentages from single live cells and also from the parent population (VE-cad^{+}CD45^{+} for type II pre-HSCs and VE-Cad^{+}CD45^{-} for pro-HSCs and type I pre-HSCs). Because we have not counted AGM cellularity when performing these experiments, we are unable to plot absolute numbers. To make the figure easier to interpret, we have now only included *Nr4a1*$^{+/+}$*;Nr4a2*$^{+/+}$ and *Nr41*$^{-/-}$*Nr4a2*$^{-/-}$ embryos. We plotted the frequencies from single live cells but have also included the results from the parent population below, for the reviewer to compare and appreciate the data variability regardless of the approach used. With either approach, we find an increase in pro-HSCs in *Nr41*$^{-/-}$*Nr4a2*$^{-/-}$ embryos, and a decrease in type II pre-HSCs.

© 2024. Published by The Company of Biologists under the terms of the Creative Commons Attribution License (https://creativecommons.org/licenses/by/4.0/).

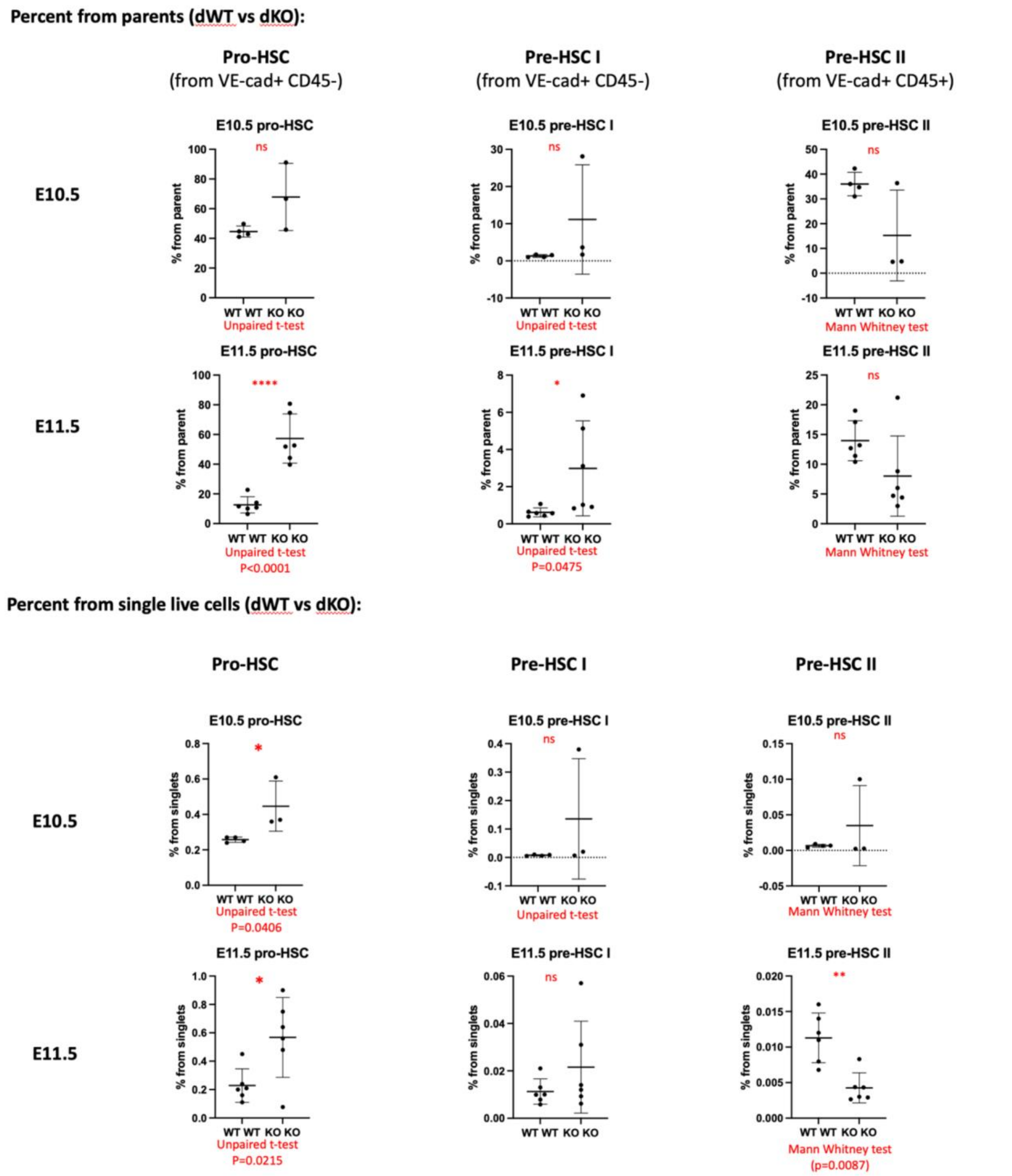

Another challenging thing to interpret in Figure 4 is the "gene dosage" effect. The authors include many different mouse strains lacking either 1 or both copies of Nr4a1 and/or Nr4a2, but then some combinations are missing and other combinations have very few and wildly different numbers of replicates. This quickly becomes impossible to interpret (and hard to perform statistics for). I would advice to increase the number of replicates across genotypes, or just stick to the ones that have enough replicates to achieve robust conclusions (WT, and the series of Nr4a1-/-, which are the last 3 columns).

As suggested, we have now only plotted *Nr4a1*$^{+/+}$;*Nr4a2*$^{+/+}$ and *Nr41*$^{-/-}$*Nr4a2*$^{-/-}$ (now Figure 3D-E).

The second biggest concern is the confusing results on Figure 5, which strikingly contrast with Figure 2. I have issue with trying to compare them side-by-side with E11.5 transplant data, as the authors attempt (in the results and discussion). First, the success of the cultures is significantly better after explant cultures in Figure 5, with most mice being able to engraft even if fewer

© 2024. Published by The Company of Biologists under the terms of the Creative Commons Attribution License (https://creativecommons.org/licenses/by/4.0/).

starting material is transplanted (0.3 EE/mouse). It is my understanding that E11 AGMs can also be processed in the same manner. It would be important to know whether the cultures also have the capacity to induce hemogenesis from Nr4a1/2 null at E11. An alternative interpretation of the data is that Nr4a null HSCs at E11.5 have increased Nfkb signaling, matured faster and have already migrated out of the AGM (something that could also fit with the E10 data). One way to test this would be to test fetal liver FACS and transplants at E11.5.

We thank the Reviewer for their insightful comment. Explant cultures induce an increase in the number of transplantable HSCs, primarily via the maturation and expansion of pre-existing pro-HSCs and pre-HSCs in the AGM (Taoudi *et al*. *Cell Stem Cell* 2008, Ganuza *et al*., *Nat Cell Biol* 2017). Our data shows that EHT has started but there is a block in maturation of pro-HSCs already at E10.5 that is maintained at E11.5. Thus, explant cultures at E11.5 would likely yield a similar result as E10.5 cultures. As we previously discussed in the manuscript, we speculate that the ectopic activation of pathways downstream of NR4A proteins *in vitro* may bypass their role and induce or resume cell proliferation and/or maturation of E10.5 HSC hemogenic endothelial precursors and/or pro-HSCs. Additionally, as we indicated to Reviewer 1 (point 3) our new data show that at E12.5 there is a lower to no difference in the number of FL HSCs, suggesting an AGM-specific defect and that HSCs have not migrated out of the AGM earlier.

In general, these seems to be a lack of attempt at describing the mechanisms and genes being regulated by Nr4a1/2. Considering these are transcriptional regulators, the most straightforward experiment would be to perform some sort of ATACseq or RNAseq experiment at E10-11. Considering the heterogeneity in clusters at E10, perhaps the best option would be to perform single-cell profiling comparing WT and Nr4a1/2-null. The most pressing question, considering the results in Figure 2, is if Nr4a1/2-null AGM lacks expression of Runx1 (placing it upstream of the EHT pathway). These could be compared with Freire et al. 2018 to see if NfkB signaling (a regulator of EHT) is also altered.

Due to the size of the EHT populations and the heterogeneity of genotypes that can be obtained from each cross, single cell RNA sequencing would require pooling at least 50 embryos of each genotype together on the same day, which would be technically very challenging and potentially not feasible. Instead, we performed spatial transcriptomic analysis of E10 *Nr4a1*$^{+/+}$;*Nr4a2*$^{+/+}$ and *Nr41*$^{-/-}$*Nr4a2*$^{-/-}$ embryos and found no differences in Runx1 expression (new figure 5C) between genotypes. We did find that *Nr41*$^{-/-}$*Nr4a2*$^{-/-}$ embryos fail to turn off Notch signaling, which explains the arrest in maturation of pro-HSCs into type II pre-HSCs. We added these data to Figure 5D and E. We also looked at NFkB signaling in HECs and IACs and found no differences between genotypes (Supplementary Fig. 3C)

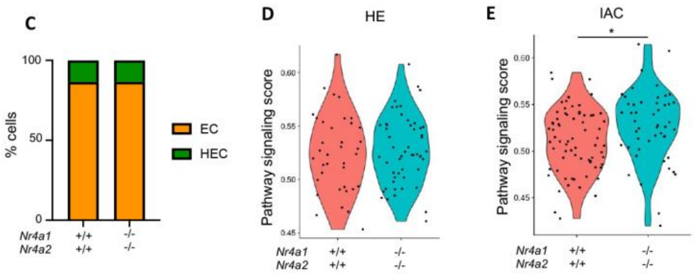

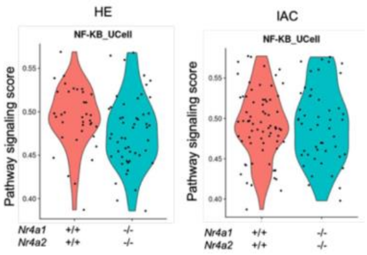

Minor concerns:

Figure S2D is not mentioned in the figure.

Added to the text. Former Fig. S2D is currently Fig S1B.

Supplementary 2A-C figures could be placed together with the main figure 2 as secondary transplant aim to demonstrate the proper functionality of donor cells. WT cells are not shown as control.

© 2024. Published by The Company of Biologists under the terms of the Creative Commons Attribution License (https://creativecommons.org/licenses/by/4.0/).

As suggested, we have placed these figures together with main figure 2. In this experiment we employed *Nr4a1*$^{-/-}$ as the internal control to compare *Nr4a1*$^{-/-}$*Nr4a2*$^{-/-}$ double knockout cells. We cannot exclude that the lack of *Nr4a1* on its own may have an effect in secondary transplantation.

Line 215 lacks Fig. 3A call and line 217 lacks Fig. 3B/C call.

References on vitelline and umbilical arteries were removed. Hence this part of the text was deleted as indicated in response to Reviewer 1 (Point 2B).

Magnification value size in Fig. 3A should be added in the main figure to understand the difference between column A and B.
Magnification values are now included in the figure.

Line 225 should specify 4A.
This was added to the text as new Fig 3D-E

Unclear why authors do not study Nr4a3 (together with 4a1 and 4a2). Is it expressed? No? This should be shown in Figure 1, perhaps.
***Nr4a3* is expressed in the AGM in VE^{+}CD45^{+} cells and it would be interesting to see if it is required in EHT in a future study. We have added expression data for *Nr4a3* to Figure 1 including qRT-PCR data to Figure 1D and expression data from # source: http://hsc.hms.harvard.edu/heat_map/export/nr4a3 to Figure 1B. In the case of scRNAseq data, Zhou et al., Nature. 2016 May 26;533(7604):487-92. (doi: 10.1038/nature17997. Supplemental Table 3; Zhou RefSeqGene.Exprssion.SingleCell) does not include expression data for *Nr4a3*, so we could not add this information. We report on Nr4a3 expression levels in lines 158-161, 171, 337.**

-Finally, to both Reviewers we would like to summarize how figures were reorganized from our previous submission in case this is helpful:

Previous Supplementary Fig. 2A-C have now been integrated in Figure 2. Supplementary Fig. 2D is now part of Supplementary Fig.1.
Supplementary Fig. 2 now contains new data showing the Chi-square results, embryo phenotypes and AGM cellularity.
Fig. 4A was now integrated into Supplementary Fig. 1, and former Fig. 4B was merged into Figure 3 and the data on vitelline and umbilical arteries was removed.
Former Figure 5 is now Figure 4, and a new Fig. 5 and Supplementary Fig. 3 were added with the new CosMx data.

Second decision letter

MS ID#: DEVELOP/2023/201957

MS TITLE: NR4A1 and NR4A2 orphan nuclear receptors regulate endothelial to hematopoietic transition in mouse hematopoietic stem cell specification

AUTHORS: Diana Sa da Bandeira, Chris D Nevitt, Felipe Segato Dezem, Maycon Marcao, Yutian Liu, Zakiya Kelley, Hannah DuBose, Ashley Chabot, Trent Hall, Claire Caprio, Victoria Okhomina, Guolian Kang, Jasmine Plummer, Shannon McKinney-Freeman, Wilson K Clements, and Miguel Ganuza

I am happy to tell you that your manuscript has been accepted for publication in Development, pending our standard publication integrity checks.

Reviewer 1

© 2024. Published by The Company of Biologists under the terms of the Creative Commons Attribution License (https://creativecommons.org/licenses/by/4.0/).

Advance summary and potential significance to field

My previous statement still holds.

Comments for the author

The reviewers have satisfactorily addressed my concerns. I have included a minor consideration in response to the authors rebuttal document. I believe that this study will be a valuable contribution to the field. Congratulations.

1. Response to 2(c) “We thank the Reviewer for their comment. Since blood circulation starts at E8.5, HSC functional defects detected in the AGM at E11.5 will likely be observed in other organs. Due to a limitation in the number of mice, we have focused on analyzing the HSC defects in the E12.5 FL, as it becomes the major hematopoietic organ from E12.5 onward. We found that the frequency of HSCs within the LSK population was unchanged with the loss of Nr4a1 and Nr4a2. However, because the frequency of LSK is lower when gated from the parent population (Lin-), we could conclude that the absolute number of HSCs (CD48-CD150+ LSK) must be lower. On the other hand, if we plot all the data from single live cells, we do not find differences in LSKs nor HSCs. Because these data are inconclusive, we have decided not to add them to the manuscript. But if the Reviewer thinks we should, we will add it as a supplementary figure and mention the unclear results. We can conclude, however, that the AGM defects we observe in Nr4a1-/-;Nr4a2-/- embryos is not due to a premature migration of HSPCs to the fetal liver.”

My opinion is that given current ideas about the possible fates of AGM region IACs (HSCs vs MPP [e.g., https://doi.org/10.1038/s41586-022-04804-z and DOI: 10.1016/j.celrep.2021.109675]), this observation is could be interesting but would require further investigation such as deconstruction of the E12.5 LSK into LT-HSC, ST-HSC, and various MPP subsets. Whether to include these additional investigations should be completely to the discretion of the authors, but perhaps this would be better suited for a future manuscript.

Reviewer 2

Advance summary and potential significance to field

The manuscript by Sa da Banderia et al. focuses on HSC emergence during endothelial to hematopoietic transition (EHT), aiming to characterize new regulators of this process. The authors identify the Nr4a family of transcription factors as putative regulators of multiple stages in HSC specification, and then test the function of these transcriptional regulators during EHT. They identify that Nr4a1 is partly redundant with Nr4a2, while the loss of both transcription factors abrogates HSC maturation during EHT, possibly through the maintenance of Notch activity within the intra-aortic clusters. In sum, the authors assign for the first time a clear role to these TFs during early HSC development, and a potential pathway that they regulate to generate or maintain mature HSC states.
Nr4a1/2 have also been identified as upregulated in inflammatory-memory and CH-mutant HSCs, so these findings might also have implications for understanding HSC aging and myeloid-bias.

Comments for the author

The revised manuscript has addressed most of the points raised by me and the other reviewer, and I am satisfied with the modifications made. I particularly think the spatial profiling dataset in the AGM comparing the wild-type and mutant Nr4a2 and the effect on Notch signaling is an interesting addition and reveals a potential mechanism for the HSC maturation blockade. I have no further comments to add.

-- Alejo E. Rodriguez-Fraticelli

© 2024. Published by The Company of Biologists under the terms of the Creative Commons Attribution License (https://creativecommons.org/licenses/by/4.0/).
